# Biazulene diimides: a new building block for organic electronic materials†
†Electronic supplementary information (ESI) available. CCDC 1483780. For ESI and crystallographic data in CIF or other electronic format see DOI: 10.1039/c6sc02504h



**DOI:** 10.1039/c6sc02504h

**Published:** 2016-07-19

**Authors:** Hanshen Xin, Congwu Ge, Xiaodi Yang, Honglei Gao, Xiaochun Yang, Xike Gao

**Affiliations:** a Key Laboratory of Synthetic and Self-Assembly Chemistry for Organic Functional Molecules , Shanghai Institute of Organic Chemistry , Chinese Academy of Sciences , 345 Lingling Road , Shanghai 200032 , China . Email: gaoxk@mail.sioc.ac.cn; b Laboratory of Advanced Materials , Fudan University , Shanghai 200433 , China; c Department of Chemistry , Shanghai University , Shanghai 200444 , China

## Abstract

Azulene, a 10-π-electron isomer of naphthalene, is a nonbenzenoid bicyclic aromatic hydrocarbon with a beautiful blue color and a large dipole moment. We present here the first class of azulene-based aromatic diimides, 2,2′-biazulene-1,1′,3,3′-tetracarboxylic diimides (BAzDIs), which comprise a 2,2′-biazulene moiety and two seven-membered imide groups. DFT calculations, thermal, optical and electrochemical properties of two BAzDI derivatives as well as single crystal analysis and the charge transport behavior were studied. The results demonstrate that BAzDIs have unique photophysical properties and are promising for organic electronic materials.

## Abstract 2

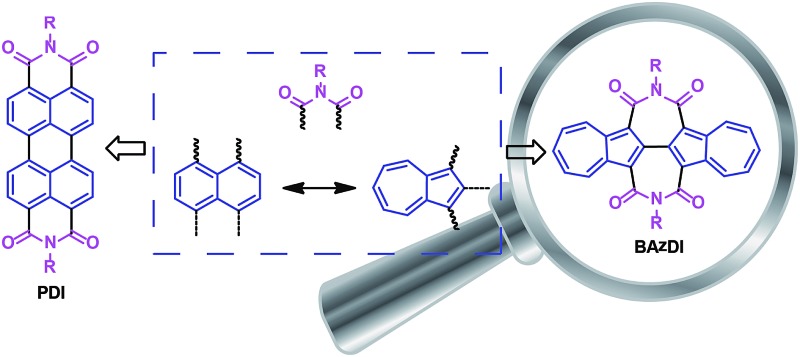
The first class of azulene-based aromatic diimides, 2,2′-biazulene-1,1′,3,3′-tetracarboxylic diimides (BAzDIs), is presented.

## Introduction

Azulene, a 10-π-electron isomer of naphthalene, is a nonalternant and nonbenzenoid bicyclic aromatic hydrocarbon, with a beautiful blue color and a dipole moment of about 1.08 D.^1^ The remarkable polarizability of azulene results from the fusion of an electron-rich five-membered ring and an electron-poor seven-membered ring, and this stable “donor–acceptor”-like resonance structure with a non-mirror-related HOMO/LUMO geometry distinguishes azulene from conventional fused benzenoids and endows azulene with unique photophysical properties.^2^ Azulene derivatives are common in nature and possess a broad range of biological activities.^3^ On the other hand, azulene and its derivatives have attracted more and more attention in materials science due to their unique optical and electronic properties.^4^ For example, azulene derivatives have been used for developing advanced organic materials, including liquid crystals,^5^ molecular switches,^6^ anion receptors/sensors,^7^ nonlinear optical materials,^8^ organic/polymeric conductors,^9^ and near-infrared resonance materials.^10^ In recent years, azulene derivatives have attracted ever increasing attention due to their successful applications in organic electronic and photovoltaic devices, such as organic field-effect transistors (OFETs),^11^ organic photovoltaics (OPVs),^12^ and perovskite solar cells.^13^

**Fig. 1:**
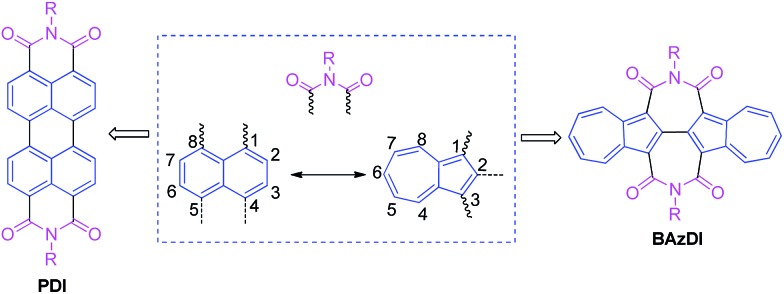
Chemical structures of PDI and BAzDI derivatives with their basic compositional units.

**Scheme 1:**
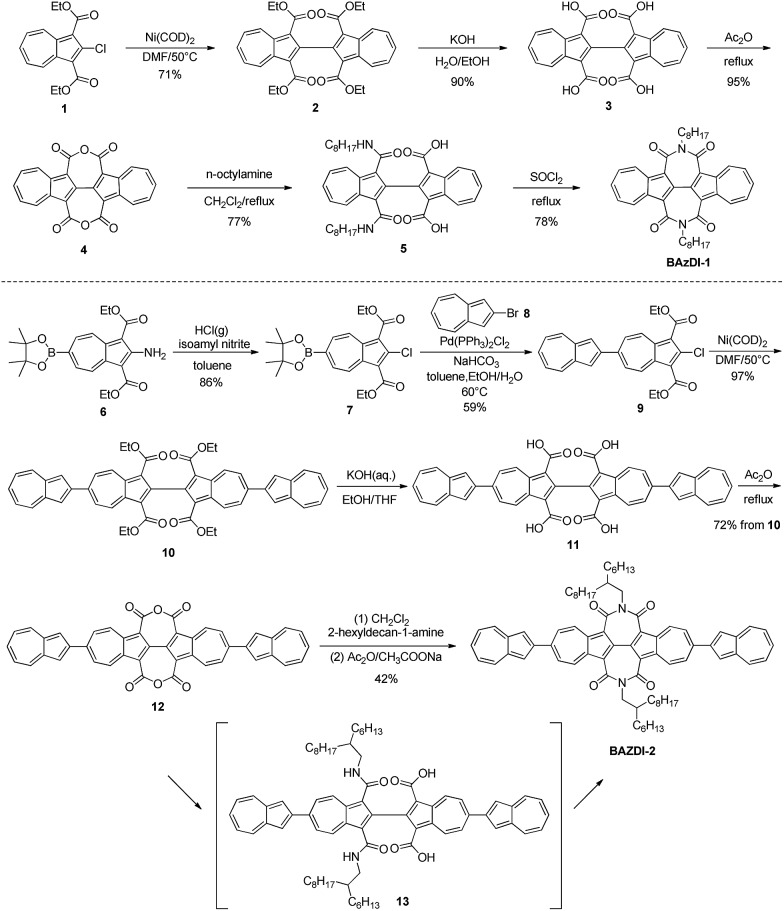
Synthesis of **BAzDI-1** and **BAzDI-2**.

Perylene diimides (PDIs), containing two 1,1′,8,8′-connected naphthalene units and two six-membered imide rings, are a class of well-known organic pigments/dyes and have played a very important role in the development of n-type organic optoelectronic materials and organic supramolecular materials.^14^ However, up to now, azulene, as an isomeric hydrocarbon of naphthalene, has not been used to construct aromatic diimides. In consideration of the aforementioned azulene's unique structural, optical and electronic features, the optoelectronic properties of azulene-based aromatic diimides would be promising and worthy of exploration. Herein, we present the first class of azulene-based aromatic diimides, 2,2′-biazulene-1,1′,3,3′-tetracarboxylic diimides (BAzDIs), which comprise a 2,2′-biazulene moiety and two seven-membered imide rings. As shown in Fig. 1, PDIs possess two 1,1′,8,8′-connected naphthalene units and two six-membered imide groups, and BAzDIs have two 2,2′-bonded azulene moieties and two seven-membered imide groups. Since naphthalene and azulene are isomers, the difference in the chemical formulas between BAzDIs and PDIs with the same R substituents is two extra hydrogen atoms for BAzDIs, which makes BAzDI derivatives interesting for the exploration of their optical/electronic properties and related applications. In this communication, we report on the first two members of the newly created BAzDI derivatives **BAzDI-1** and **BAzDI-2** (Scheme 1) and their preliminary application in organic electronic devices; **BAzDI-1** represents the parent molecular backbone of BAzDIs with two *n*-octyl substituents, and **BAzDI-2** is a 6,6′-derived molecule of BAzDI (R = 2-hexyldecyl) with two terminal 2-position-connected azulene units.

## Results and discussion

The synthetic routes of **BAzDI-1** and **BAzDI-2** are shown in Scheme 1. We first tried to synthesize tetracarboxylate **2** (ref. 16) from **1** (ref. 15) by a copper catalyzed Ullmann coupling reaction based on a literature method,^16^ but it failed. After some exploration, we found that the self-coupling of **1** could be performed efficiently by using Ni(COD)_2_ as a catalyst in DMF at 50 °C to give **2** in 71% yield. Hydrolysis of **2** provided acid **3** (ref. 16) in 90% yield. Then we treated **3** with refluxing acetic anhydride, affording the intermediate dianhydride **4** (ref. 16) in 95% yield. Carbamoyl **5** was obtained in 77% yield by adding *n*-octylamine to the solution of **4** in refluxing dichloromethane. The imidization reaction of **5** in refluxing thionyl chloride afforded *N*,*N*′-bis(*n*-octyl)-2,2′-biazulene-1,1′,3,3′-tetracarboxdiimide **BAzDI-1** with a yield of 78%. Chlorination of **6** (ref. 17) produced dicarboxylate **7** in 86% yield. Compound **9** was prepared in 59% yield by a Suzuki cross-coupling reaction of **7** with 2-bromoazulene **8**.^18^ The intermediate **9** then underwent a similar self-coupling reaction to **1**, giving the key precursor **10** in 94% yield. Hydrolysis of **10** gave acid **11**, which was not purified and directly underwent a condensation reaction in refluxing acetic anhydride, affording dianhydride **12** with an overall two-step yield of 72%. Compound **12** was reacted with 2-hexyldecylamine in refluxing dichloromethane, giving the intermediate carbamoyl derivative **13**. Compound **13** was not isolated and directly underwent an imidization reaction with acetic anhydride and sodium acetate to give *N*,*N*′-bis(2-hexyldecyl)-2,6′:2′,2′′:6′′,2′′′-quaterazulene-1′,1′′,3′,3′′-tetracarboxdiimide **BAzDI-2** in 42% overall yield from **12**.

The chemical structures of **BAzDI-1** and **BAzDI-2** were fully characterized using ^1^H and ^13^C NMR spectroscopy, high-resolution mass spectrometry and FT-IR spectrometry as well as elemental analysis. At room temperature, **BAzDI-1** and **BAzDI-2** are highly soluble in most common organic solvents such as CH_2_Cl_2_, CHCl_3_, THF, and toluene. Thermogravimetric analysis (TGA) measurements demonstrate that **BAzDI-1** and **BAzDI-2** are thermally stable with decomposition temperatures of 376 °C and 377 °C, respectively (Fig. S1a and S1b†). As shown in Fig. S1c and S1d,† both **BAzDI-1** and **BAzDI-2** display two couples of reversible endothermic and exothermic peaks in differential scanning calorimetry (DSC) cycles; the former peaks are relatively weak with low enthalpy, demonstrating some solid–solid phase transition, and the latter peaks suggesting melting and crystallization are strong and sharp with high enthalpy. Their melting points revealed by DSC analyses are 205 °C and 282 °C, respectively.

**Fig. 2:**
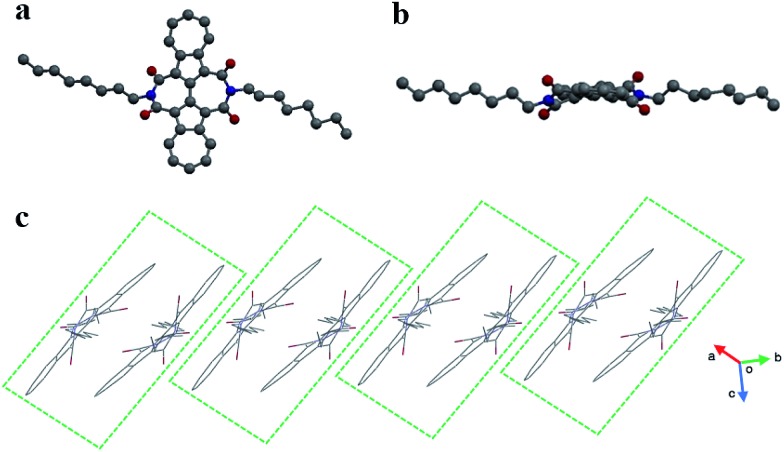
The molecular structure (a: top view, b: side view) and molecular packing (c) of **BAzDI-1** in a single crystal.

A single crystal of **BAzDI-1** obtained by a diffusion method using dichloromethane and methanol solvents showed a twisted molecular backbone as illustrated in Fig. 2. The dihedral angle of two azulene rings is about 17.8°, and the dihedral angles between 2,2′-biazulene and two respective imide rings are around 27.7° and 29.6°, demonstrating that the imide rings deviate from the plane of the 2,2′-biazulene. As shown in Fig. S2,† the C(5)–C(6) bond distance (1.43 Å) is shorter than the normal C(sp^2^)–C(sp^2^) bond (1.48 Å), indicating good π-electron delocalization between the two azulene units. The electron-deficient seven-membered ring of one azulene unit overlaps with the electron-rich five-membered ring of the adjacent azulene moiety to form the molecule dimers with an interplanar π–π stacking distance of about 3.53 Å. These **BAzDI-1** dimers form a slipped one-dimensional (1D) packing motif in the crystal with short intermolecular atom–atom contacts of about 3.15–3.38 Å (Fig. 2c). This 1D π–π stacking arrangement is expected to favor charge transport and impact on other physical properties of **BAzDI-1** and its derivatives.

**Fig. 3:**
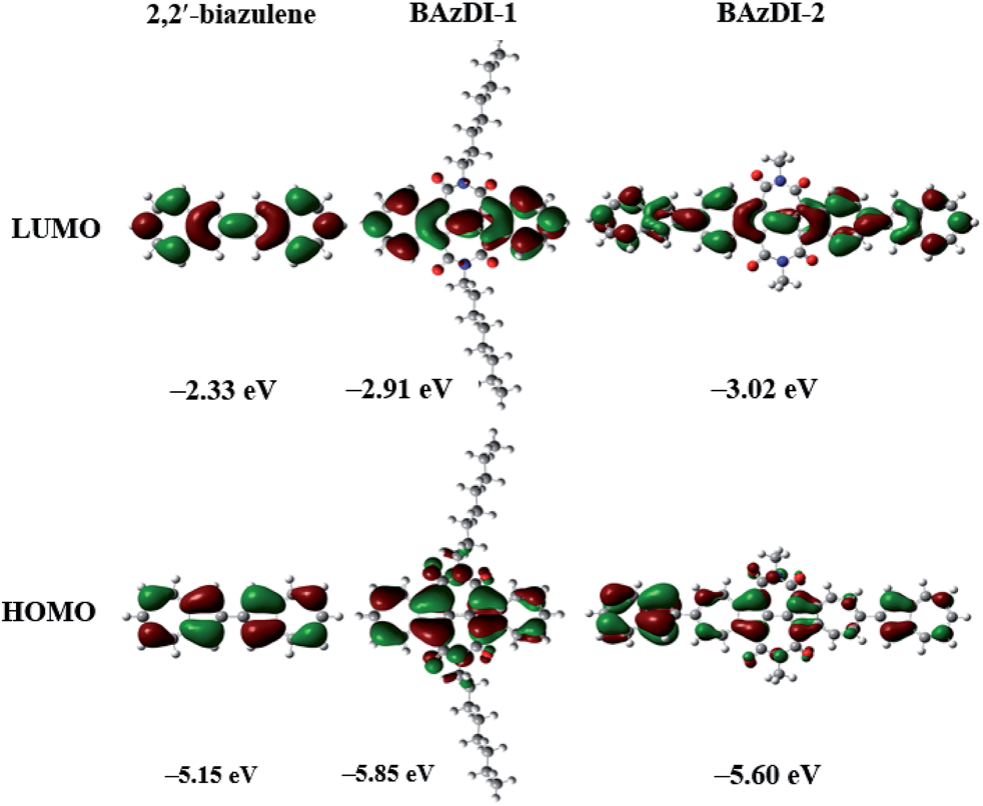
Frontier molecular orbitals and their energies for 2,2′-biazulene, **BAzDI-1**, and a *N*,*N*′-bis(methyl)-substituted model molecule for **BAzDI-2**, obtained by DFT calculations.

To evaluate the positions and energies of frontier molecular orbitals (FMO) for **BAzDI-1** and **BAzDI-2**, as well as 2,2′-biazulene, Density Functional Theory (DFT) calculations were carried out at the B3LYP/6-31G(d,p) level using the Gaussian 09 program. Alkyl chains were replaced by methyl groups for **BAzDI-2** to reduce the time required for the calculations. As shown in Fig. 3, for all three molecules, the density distribution of the LUMO is spread over the whole molecule backbone except the imide groups of **BAzDI-1** and **BAzDI-2**. However, for their HOMO, there is no orbital density located on the 2 and 6 positions of all the azulene units of the molecular backbone. Notably, the HOMO density distribution is mainly located on one of two terminal azulene rings of **BAzDI-2**, whereas the LUMO density distribution is well-distributed over the whole molecular carbon backbone. This biased distribution of HOMO density makes hole transport unfavored, and also implies considerable intramolecular charge transfer. **BAzDI-1** can be regarded as the product obtained by the addition of two seven-membered imide rings to 2,2′-biazulene at the 1,1′ and 3,3′ positions. This structural change greatly affects the FMO energies; the HOMO and LUMO energies of **BAzDI-1** shift downward *versus* those of 2,2′-biazulene (HOMO: –5.15 eV; LUMO: –2.33 eV; HOMO–LUMO gap: 2.82 eV), giving relatively lower FMO energies (HOMO: –5.85 eV; LUMO: –2.91 eV) and a slightly broader HOMO–LUMO gap (2.94 eV). This downward-shift in FMO energies may be attributed to the electron-withdrawing ability of the two imide groups. Compared to the FMO energies of **BAzDI-1**, **BAzDI-2** (HOMO: –5.60 eV; LUMO: –3.02 eV; HOMO–LUMO gap: 2.58 eV) presents a higher HOMO energy, a relatively lower LUMO energy, and a narrower HOMO–LUMO gap induced by the extension of the π-system.

**Fig. 4:**
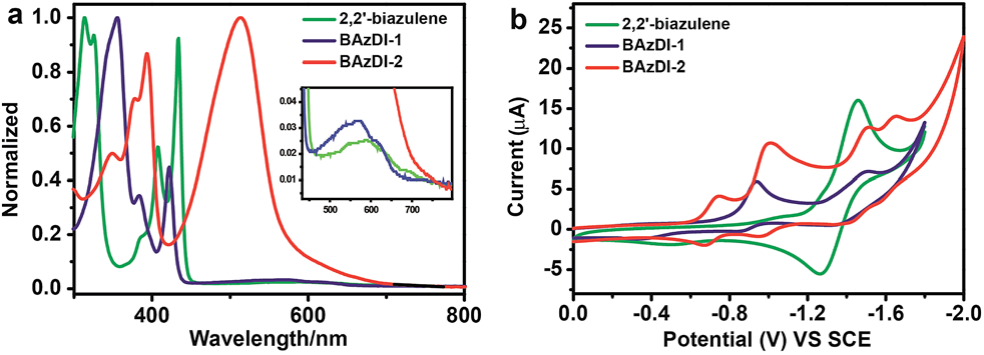
(a) UV-vis spectra of 2,2′-biazulene, **BAzDI-1** and **BAzDI-2** in dichloromethane with magnified long-wavelength absorptions inserted. (b) Cyclic voltammograms of 2,2′-biazulene, **BAzDI-1** and **BAzDI-2** in dichloromethane (0.1 M Bu_4_^+^NPF_6_^–^ as supporting electrolyte; SCE as reference electrode; scan rate of 100 mV s^–1^).

The UV-vis absorption spectra of **BAzDI-1** and **BAzDI-2** in dichloromethane were measured and compared with that of the parent 2,2′-biazulene to investigate the optical properties (Fig. 4a). The data are summarized in Table 1. 2,2′-Biazulene exhibited a strong absorption at 434 nm (*ε* = 82 600 M^–1^ cm^–1^), derived from the S_0_ → S_2_ transition, and a weak absorption at about 600 nm (*ε* = 3300 M^–1^ cm^–1^) that corresponds to the S_0_ → S_1_ transition.^2^ In comparison with those of 2,2′-biazulene, both the former peak and the long-wavelength absorption of **BAzDI-1** showed blue-shifts to 422 nm (*ε* = 46 500 M^–1^ cm^–1^) and 572 nm (*ε* = 5800 M^–1^ cm^–1^), respectively. These changes can be ascribed to the fact that the 2,2′-biazulene unit of **BAzDI-1** is twisted due to the two attached imide rings that are out of the plane of the 2,2′-biazulene, as revealed by DFT calculations (Fig. S3†) and X-ray crystallography (Fig. 2). In contrast, with the addition of two azulene units at the 6 and 6′ positions of **BAzDI-1**, **BAzDI-2** displayed a broad and strong absorption from about 400 to 700 nm (absorption peak at about 513 nm, *ε* = 127 300 M^–1^ cm^–1^), which is different from the very weak end absorptions of 2,2′-biazulene and **BAzDI-1** (Fig. 4a) and can be assigned to the intramolecular charge transfer as well as the S_0_ → S_1_ transition. The absorption spectra of **BAzDI-1** and **BAzDI-2** in thin films are shown in Fig. S4.† For **BAzDI-1**, the thin film absorption at 592 nm is ascribed to the S_0_ → S_1_ transition, which is red shifted relative to the solution end absorption (at 572 nm). Interestingly, in comparison with the solution absorption, the thin film absorption for **BAzDI-2** showed a broader absorption band at about 511 nm with a shoulder peak at about 570 nm, which may indicate that strong molecular packing is present in the film. Optical band gaps for 2,2′-biazulene, **BAzDI-1** and **BAzDI-2** estimated from the onset of the end absorptions in the solution are 1.79 eV, 1.86 eV and 1.69 eV, respectively, and the trends of these optical band gap values are consistent with the HOMO–LUMO gap values estimated from the DFT calculations. It should be noted that the molecular formulas of the molecular backbones of BAzDIs and PDIs (Fig. 1) differ by two hydrogen atoms. However, the absorption and emission spectra as well as the color in solution and the solid state for **BAzDI-1** are quite different from those of a PDI derivative with alkyl chain substituents (Fig. S5†), and **BAzDI-1** has relatively higher LUMO energy and a narrower band gap.

**Table 1:** Optical, electrochemical and DFT calculation data for 2,2′-biazulene, **BAzDI-1** and **BAzDI-2**

Compound	*λ*_max_ (nm)		LUMO^*a*^ (eV)	HOMO^*b*^ (eV)	*E*_g_ ^*c*^ (eV) sol	LUMO^*d*^ (eV)	HOMO^*d*^ (eV)
	Sol	Film					
2,2′-Biazulene	434, 598		–3.08	–4.87	1.79	–2.33	–5.15
**BAzDI-1**	422, 572	428, 592	–3.53	–5.39	1.86	–2.91	–5.85
**BAzDI-2**	513	511, 570	–3.74	–5.43	1.69	–3.02	–5.60

^*a*^Estimated from the equation LUMO = –4.44 – *E*_red1_^1/2^ (calibration by ferrocene).

^*b*^Estimated from HOMO = LUMO – *E*_g_.

^*c*^Estimated from the edge of end absorption.

^*d*^Estimated from DFT calculations.

As shown in Fig. 4b, cyclic voltammetry (CV) for 2,2′-biazulene exhibits a reversible reduction process with a half-wave reductive potential (*E*_red_^1/2^) of –1.36 V. In comparison with 2,2′-biazulene, **BAzDI-1** presents two quasi-reversible redox waves with a first half-wave reductive potential (*E*_red1_^1/2^) of –0.91 V, indicating that the introduction of imide groups endows **BAzDI-1** with better oxidative ability. On the other hand, **BAzDI-2** displays four quasi-reversible reduction processes with *E*_red1_^1/2^ at about –0.70 V. The LUMO levels of 2,2′-biazulene, **BAzDI-1** and **BAzDI-2**, estimated by CV measurements, are –3.08 eV, –3.53 eV and –3.74 eV, respectively, which is consistent with the DFT results although there are large deviations of about 0.7 eV. The relatively lower LUMO levels of **BAzDI-1** and **BAzDI-2** enable them to be potential n-type semiconducting materials for organic optoelectronic devices.

**Fig. 5:**
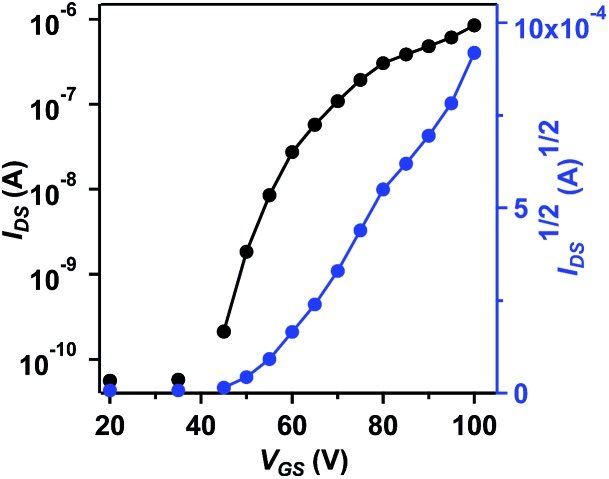
Transfer curve of a thin-film FET device based on **BAzDI-2**, annealed at 120 °C and *V*_DS_ = 60 V.

The charge transport properties of **BAzDI-2** were investigated using field-effect transistors (FETs). Thin films of **BAzDI-2** were spin-coated onto octadecyltrichlorosilane (OTS)-treated Si/SiO_2_ substrates. Au source/drain electrodes were deposited on the active layer to afford a bottom-gate top-contact device configuration. The FET devices were tested in a glovebox with a nitrogen atmosphere. As shown in Fig. 5, all the devices exhibited n-type semiconductor characteristics, which correlates well with the aforementioned DFT and CV results. The data for FET device performance for **BAzDI-2** are summarized in Table S2.† The electron mobility gradually improved with increased thin film annealing temperature. Thin film FET devices based on **BAzDI-2** (annealed at 120 °C) demonstrated an electron mobility of 1.5 × 10^–2^ cm^2^ V^–1^ s^–1^, and a current on/off ratio of 10^4^ to 10^5^. As shown in Fig. S6,† X-ray diffraction (XRD) patterns of thin films of **BAzDI-2** annealed at different temperatures are presented. The as-spun thin films of **BAzDI-2** showed no appreciable diffraction peaks, suggesting that the films were disordered and the corresponding device performance was relatively low. When annealed at 80 °C and 120 °C, the thin films of **BAzDI-2** exhibited first order reflection, implying a degree of crystallinity, which was consistent with the relatively higher device performance. Atomic force microscopy (AFM) images of thin films of **BAzDI-2** annealed at different temperatures are provided in Fig. S7,† and the crude featureless thin-film morphology may be one of the reasons for the relatively lower preliminary device performance. Although the preliminary device performance of **BazDI-2** is not high enough, in view of the completely new structure of BAzDIs, further device optimisation and chemical modifications on the molecular backbone of BAzDIs would be expected to achieve high device performance and new molecular functions.

## Conclusions

In summary, we have reported the first class of azulene-based aromatic diimides, 2,2′-biazulene-1,1′,3,3′-tetracarboxylic diimides (BAzDIs), which is a new class of organic dyes. The synthesis, X-ray crystal structure, DFT calculations, absorption spectra and electrochemical properties as well as the charge transport characteristics were investigated. The results demonstrate that BAzDIs exhibit unique chemical structures and frontier molecular orbitals, with relatively higher LUMO energies as well as narrower band gaps than those of corresponding PDIs. We believe that BAzDIs would be an interesting building block for organic electronic materials and could also play an important role in exploiting novel advanced materials with unique structures and functions. Chemical modifications of the molecular backbone of BAzDIs and their application in organic optoelectronic devices are currently underway in our lab.
