# Supplementary material for: Biazulene diimides: a new building block for organic electronic materials
Source: Chem Sci. 2016 Jul 19;7(11):6701–5. doi: 10.1039/c6sc02504h (PMC5355808; doi:10.1039/c6sc02504h)
Supplement: Supplementary file 1 [file SC-007-C6SC02504H-s001.pdf]

Supporting information

Biazulene Diimides: A New Building Block for Organic Electronic Materials.

*Hanshen Xin, Congwu Ge, Xiaodi Yang, Honglei Gao, Xiaochun Yang, and Xike Gao**

Contents:

1. Materials and general methods. S2
2. Synthesis of compounds **1–12**, BAzDI-1 and BAzDI-2 S2-S9
3. TGA and DSC curves for BAzDI-1 and BAzDI-2. S10
4. X-ray Crystallographic Structure for BAzDI-1. S10-S11
5. The geometries of 2,2′-biazulene, BAzDI-1 and BAzDI-2 obtained by DFT Calculations. S12
6. UV-Vis spectra of BAzDI-1 and BAzDI-2 in thin film. S12
7. Spectra and Color images of BAzDI-1 and PDI. S13
8. Characteristics of OFET devices based on BAzDI-2. S14
9. XRD and AFM measurements for BAzDI-2. S14
10. NMR, MS and IR spectra. S15-S45

Experimental Section

1. Materials and General Methods.

Bis(1,5-cyclooctadiene)nickel(0)， Bis(triphenylphosphine)palladium(II) dichloride were purchased from Aldrich and used without further purification. Other reagents were obtained commercially and used as received. ^{1}H NMR (300 MHz or 400 MHz) and ^{13}C NMR (100 MHz) spectra were measured on Varian Mercury (300 MHz and 400 MHz) instruments. Elemental analyses were performed on an ElementarVario EL III elemental analyzer. Mass spectra (DART-FT，ESI-FT and MALDI-FT) were carried out on a Thermo Fisher Scientific LTQ FT Ultra Mass Spectrometer. Optical absorption spectra were measured on a U-3900 UV-vis spectrophotometer. Fluorescence spectra were measured on a HITACHI F-2700 fluorescence spectrophotometer (for room temperature measurements) or Perkin-Elmer spectrofluorometer LS 55 (for –198 °C measurements). TGA measurements were conducted on a TGA Q500 instruments under a dry nitrogen flow at a heating rate of 10 °C/min, heating from room temperature to 500 °C or 600 °C. DSC analyses were performed on a DSC Q2000 instruments under a dry nitrogen flow at a heating rate of 5 °C/min, heating from –30 °C to 300 °C for BAzDI-1 and from –25 °C to 330 °C for BAzDI-2. Electrochemical measurements was carried out on a CHI610D instruments in a conventional three-electrode cell with a platinum button working electrode, a platinum wire counter electrode, and a saturated calomel electrode (SCE) reference electrode. Melting point were measured on an SGW X-4 microscopic melting point apparatus**.**

2. Synthesis

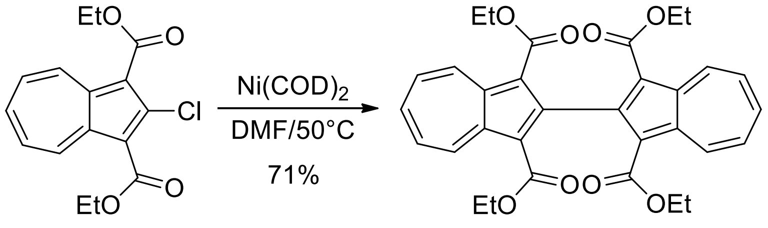

1 2

Tetraethyl 2,2'-biazulene-1,1',3,3'-tetracarboxylate 2： A mixture of Diethyl 2-chloroazulene-1,3-dicarboxylate (920 mg, 3 mmol) **1** and Ni(COD)$_2$ (454 mg, 1.65 mmol) was dissolved in DMF (10 mL) under nitrogen atmosphere. The reaction mixture was heated at 50 °C for 6 h. It was then poured into water and extracted with dichloromethane. The combined organic phases was washed with water and dried over Na_2SO_4. After concentrated under reduced pressure, the residue was purified by column chromatography on silica gel with dichloromethane/ hexane (2:1) to give product as red crystals (572 mg, 71% yield). ^{1}H NMR (400 MHz, $CDCl_3$), δ (ppm): 9.81 (d, J = 10.1 Hz, 4H), 7.92 (t, J = 9.7 Hz, 2H), 7.74 (dd, J = 10.1 Hz, 9.7 Hz, 4H), 3.89 (q, J = 7.0 Hz, 8H), 0.52 (t, J = 7.0 Hz, 12H). ^{13}C NMR (100 MHz, $CDCl_3$), δ (ppm): 165.2, 155.3, 143.3, 139.6, 138.2, 130.4, 116.1, 59.3, 13.3. MS (MALDI) m/z: 542.0 $(M)^+$. HRMS (DART-FT) (m/z): $(M+H)^+$ Calcd for $C_{32}H_{31}O_8$ 543.2013; Found, 543.2000.

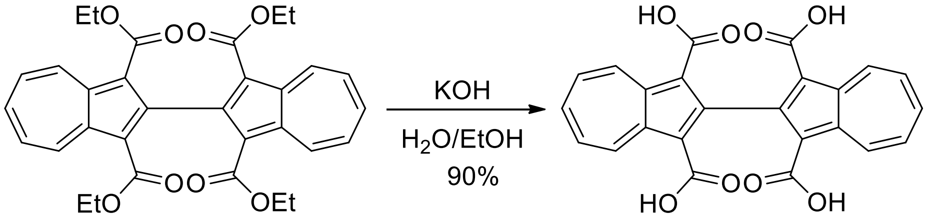

2,2'-biazulene-1,1',3,3'-tetracarboxylic acid 3: A mixture of tetraethyl 2,2'-biazulene-1,1',3,3'-tetracarboxylate **2** (542 mg, 1.0 mmol) , EtOH (16 mL) and 12 M KOH aq. (1 mL) was refluxed for 4 h. The mixture was diluted with water and filtered to remove insoluble materials. The filtrate was then acidified with 2 M HCl and red crystals separated out were collected by filtration to give product as a red solid (386 mg, 90% yield). ^{1}H NMR (300 MHz, DMSO-d_6) δ (ppm): 11.86 (s, 4H), 9.71 (d, J = 10.3 Hz, 4H), 8.08 (t, J = 9.9 Hz, 2H), 7.86 (dd, J = 10.3 Hz, 9.9 Hz 4H). ^{13}C NMR (100 MHz, DMSO-d_6) δ 166.0, 155.6, 142.5, 139.9, 137.6, 130.1, 116.5. MS (MALDI) m/z: 452.4 $(M+Na)^+$. HRMS (DART-FT) (m/z): $(M+H)^+$ Calcd for $C_{24}H_{14}O_8Na$ 453.0581; Found, 453.0577.

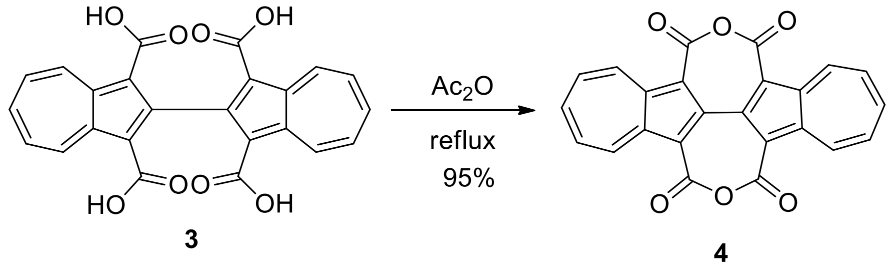

2,2'-biazulene-1,1',3,3'-tetracarboxylic dianhydride 4: A mixture of 2,2'-biazulene-1,1',3,3'-tetracarboxylic acid **3** (430 mg, 1.0 mmol) and acetic anhydride (5 mL) was refluxed for 2 h. Then the mixture was filtrated to give product as a red solid (374 mg, 95% yield). FT-IR (KBr, cm^{-1}) ν 3079.8, 2396.4, 1705.1, 1686.1, 1579.7, 1453.1, 1431.0, 1381.8, 1368.0, 1327.3, 1299.1, 1240.9, 1159.9, 1132.9, 1091.3, 1067.7, 1016.8, 956.4, 884.7, 857.3, 760.7, 687.8, 592.7, 563.2, 476.7, 417.7. Anal. Calcd for $C_{24}H_{10}O_6$: C, 73.10; H, 2.56. Found: C, 73.00; H, 2.60.

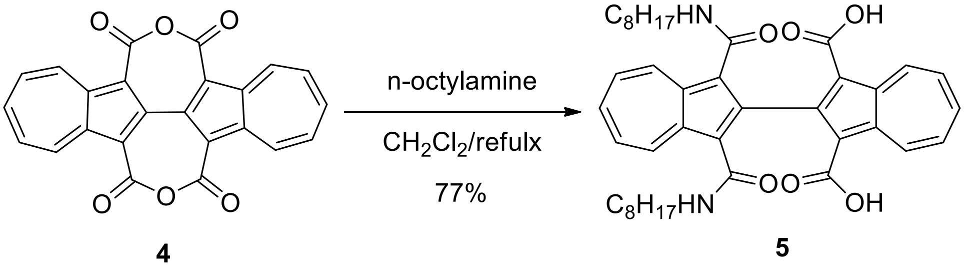

1',3'-bis(octylcarbamoyl)-2,2'-biazulene-1,3-dicarboxylic acid 5: A solution of n-octylamine solution (113 mg, 0.88 mmol) in 5 mL of dichloromethane was added dropwise to the solution of 2,2'-biazulene-1,1',3,3'-tetracarboxylic dianhydride **4** (160 mg, 0.4 mmol) in 10 mL of dichloromethane. The reaction was stirred under reflux for 4h. Upon removal of solvent, the residue was purified by column chromatography with dichloromethane/ ethanol (20:1) to give product as a purple solid (204 mg, 77% yield). ^{1}H NMR (400 MHz, DMSO-d_6), δ (ppm): 9.55 (d, J = 10.0 Hz, 2H), 9.02 (d, J = 9.8 Hz, 2H), 8.20 (t, J = 9.8 Hz, 1H), 7.95 (t, J = 10.0 Hz, 1H), 7.95 (dd, J = 10.0 Hz, 10.0 Hz, 2H), 7.60 (dd, J = 10.0 Hz, 9.8 Hz, 2H), 6.89 (t, J = 5.6 Hz, 2H), 2.99 (m, J = 4H). 1.20-1.13 (m, 4H), 1.06-0.98 (m, 4H), 0.86-0.59 (m, 22H). ^{13}C NMR (100 MHz, $CDCl_3$), δ (ppm): 166. 6, 166.4, 146.5, 143.7, 142.6, 141.2, 140.1, 137.6, 130.7, 126.6, 120.3, 117.8, 39.7, 31.7, 29.0, 28.8, 28.7, 26.4, 22.5, 14.1. MS (MALDI) m/z: 675.3 $(M+Na)^+$. HRMS (MALDI-FT) (m/z): $(M+H)^+$ Calcd for $C_{40}H_{49}O_6N_2$ 653.3585;

Found, 653.3586.

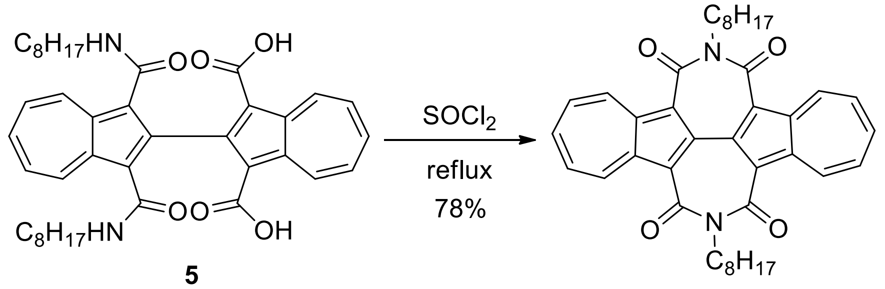

5 **BAzDI-1**

***N,N'*-bis(n-octyl)-2,2'-biazulene-1,1',3,3'- tetracarboxdiimide BAzDI-1:** A solution of 1',3'-bis(octylcarbamoyl)-2,2'-biazulene-1,3-dicarboxylic acid **5** (90 mg, 0.14 mmol) in 5 mL of thionyl chloride was refluxed for 3 h. Then all thionyl chloride was removed under vaccum. The residual oil was purified by column chromatography using dichloromethane/hexane (2:1) as eluent to provide product as a green solid (66 mg, 78% yield). M.p. =205 °C. ^{1}H NMR (400 MHz, $CDCl_3$) δ (ppm): 9.83 (d, J = 10.6 Hz, 4H), 8.04 (t, J = 9.3 Hz, 2H), 7.82 (dd, J = 10.6 Hz, 9.3 Hz, 4H), 4.40 (t, J = 7.4 Hz, 4H), 1.86 (m, 4H), 1.47 – 1.26 (m, 20H), 0.86 (t, J = 6.6 Hz, 6H). ^{13}C NMR (100 MHz, $CDCl_3$) δ (ppm): 165.9, 143.9, 142.2, 140.7, 136.7, 131.3, 118.6, 46.6, 31.8, 29.4, 29.3, 29.0, 27.4, 22.7, 14.1. FT-IR (KBr, cm^{-1}) ν 2919.2, 2851.1, 2359.5 1647.1, 1614.4, 1452.0, 1420.4, 1389.7, 1317.3, 1296.4, 1253.2, 1167.6, 1138.4, 1034.4, 959.3, 886.7, 855.3, 806.8, 772.9, 746.6, 734.5, 707.3, 684.1, 612.8, 569.9. Anal. Calcd for $C_{40}H_{44}O_4N_2$: C, 77.89; H, 7.19; N, 4.54. Found: C, 77.74; H, 7.24; N, 4.55. MS (MALDI) m/z: 616.2 $(M)^+$. HRMS (DART-FT) (m/z): $(M+H)^+$ Calcd for $C_{40}H_{45}O_4N_2$ 617.3374; Found, 617.3362.

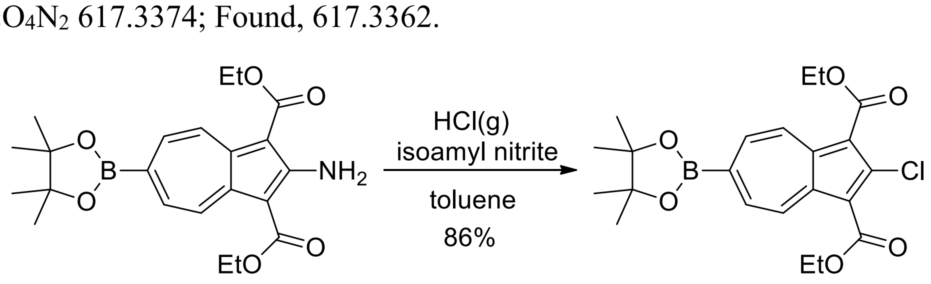

6 **7**

Diethyl 2-chloro-6-(4,4,5,5-tetramethyl-1,3,2-dioxaborolan-2-yl)azulene-1,3-dicarboxylate 7: Dry hydrogen chloride gas passed through a solution of Diethyl 2-amino-6-(4,4,5,5-tetramethyl-1,3,2-dioxaborolan-2-yl)azulene-1,3-dicarboxylate **6**

(1.24 g, 3.0 mmol) in toluene (75 mL) at 5 °C. After isoamyl nitrite (1.76 g, 15 mmol) was added dropwise, the mixture was stirred and green precipitates began to separate out. It was then left to sit at room temperature to react for 72 h until the color changed to dark red. The mixture was poured into water (150 mL) and extracted with toluene. The organic phase was dried over Na_2SO_4 and concentrated under reduced pressure. The residue was purified by column chromatography with hexane/ethyl acetate (5:1) to give product as Reddish violet crystals (1.12 g, 86% yield). ^{1}H NMR (400 MHz, $CDCl_3$) δ (ppm): 9.44 (d, J = 10.2 Hz, 2H), 8.21 (d, J = 10.2 Hz, 2H), 4.47 (d, J = 7.1 Hz, 4H), 1.46 (t, J = 7.1 Hz, 6H). ^{13}C NMR (100 MHz, $CDCl_3$) δ (ppm): 164.2, 144.8, 142.7, 136.8, 115.0, 85.2, 60.6, 24.9, 14.4. MS (MALDI) m/z: 433.1 $(M+H)^+$. HRMS (DART-FT) (m/z): $(M+H)^+$ Calcd for $C_{22}H_{27}O_6BCl$ 432.1620; Found, 432.1616.

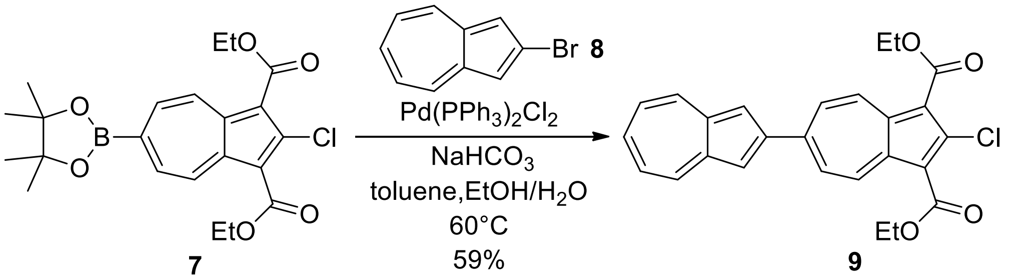

Diethyl 2'-chloro-2,6'-biazulene-1',3'-dicarboxylate 9: A mixture of $Pd(PPh_3)_2Cl_2$ (140 mg, 0.2 mmol), 2-bromoazulene **8** (414 mg, 2.0 mmol), Diethyl 2-chloro-6-(4,4,5,5-tetramethyl-1,3,2-dioxaborolan-2-yl)azulene-1,3-dicarboxylate **7** (1.56 g, 3.6 mmol), 2M $NaHCO_3$ aq. (4 mL) in toluene (8 mL) and EtOH (4 mL) was reacted at 60 °C for 2 h under nitrogen atmosphere. The mixture was poured into water (50 mL) and extracted with dichloromethane. The organic phase was dried over Na_2SO_4 and concentrated under reduced pressure. The residual was purified by column chromatography using dichloromethane/hexane (2:1) as eluent to provide product as dark green solid (510 mg, 59% yield). ^{1}H NMR (300 MHz, DMSO-d_6) δ (ppm): 9.44 (d, J = 11.1 Hz, 2H), 8.71 (d, J = 11.1 Hz, 2H), 8.50 (d, J = 9.6 Hz, 2H), 8.08 (s, 2H), 7.73 (t, J = 9.8 Hz, 1H), 7.32 (dd, J = 9.8 Hz, 9.6 Hz, 2H), 4.44 (q, J = 7.1 Hz, 4H), 1.42 (t, J = 7.1 Hz, 6H). ^{13}C NMR (100 MHz, $CDCl_3$) δ (ppm): 164.3, 149.9, 148.4, 142.9, 141.3, 140.5, 138.7, 138.2, 136.9, 130.8, 124.5, 116.3, 115.3, 60.6, 14.5. MS (MALDI) m/z: 433.1 $(M+H)^+$. HRMS (DART-FT) (m/z): $(M+H)^+$

Calcd for $C_{26}H_{22}O_4Cl$ 433.1201; Found, 433.1201.

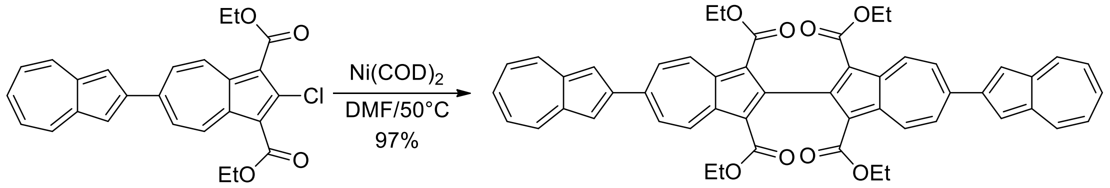

9 **10**

Tetraethyl 2,6':2',2'':6'',2'''-quaterazulene-1',1'',3',3''-tetracarboxylate 10: A mixture of Diethyl 2'-chloro-2,6'-biazulene-1',3'-dicarboxylate **9** (1.68 g, 3.7 mmol) and $Ni(COD)_2$ (1.0 g, 3.7 mmol) was dissolved in DMF (12 mL) under nitrogen atmosphere. The reaction mixture was heated at 50 °C for 6 h. It was then poured into water and extracted with dichloromethane. The combined organic phases was washed with water and dried over Na_2SO_4. After concentrated under reduced pressure, the residue was purified by column chromatography on silica gel with dichloromethane/ hexane (8:1) to give product as dark green crystals (1.49 g, 97% yield). ^{1}H NMR (300 MHz, $CDCl_3$) δ (ppm): 9.86 (d, *J* = 10.8 Hz, 4H), 8.43 (d, *J* = 10.8 Hz, 4H), 8.41 (d, *J* = 9.6 Hz, 4H), 7.86 (s, 4H), 7.62 (t, *J* = 10.0 Hz, 2H), 7.23 (dd, *J* = 10.0 Hz, 9.6 Hz, 4H), 3.97 (q, *J* = 7.0 Hz, 8H), 0.64 (t, *J* = 7.0 Hz, 12H). ^{13}C NMR (100 MHz, $CDCl_3$) δ (ppm): 165.3, 155.4, 151.1, 147.8, 142.3, 141.5, 138.4, 138.0, 137.2, 130.4, 124.5, 116.5, 116.4, 59.4, 13.4. MS (MALDI) m/z: 817.1 $(M+Na)^+$. HRMS (DART-FT) (m/z): $(M+H)^+$ Calcd for $C_{52}H_{43}O_8$ 795.2952; Found, 795.2945.

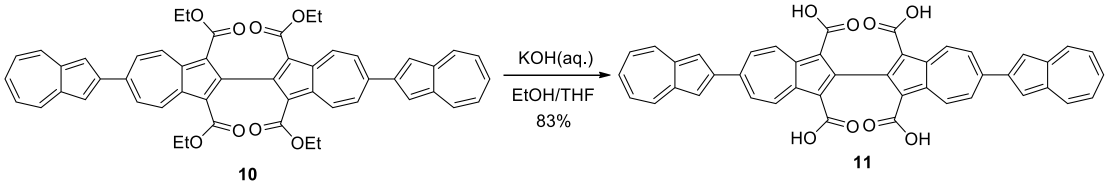

10 **11**

2,6':2',2'':6'',2'''-quaterazulene-1',1'',3',3''-tetracarboxylic acid 11: A mixture of tetraethyl 2,6':2',2'':6'',2'''-quaterazulene-1',1'',3',3''-tetracarboxylate (794 mg, 1.0 mmol) , THF (12 mL), EtOH (16 mL) and 12 M KOH aq. (1 mL) was refluxed for 24 h. The mixture was diluted with water and filtered to remove insoluble materials. The filtrate was then acidified with 2 M HCl and crystals separated out were collected by filtration to give product as a brown solid (636 mg, 83% yield). MS (ESI) m/z: 681.2 $(M–H)^-$. HRMS (ESI Negative) (m/z): $(M–H)^-$ Calcd for $C_{44}H_{25}O_8$ 681.1555; Found,

681.1544.

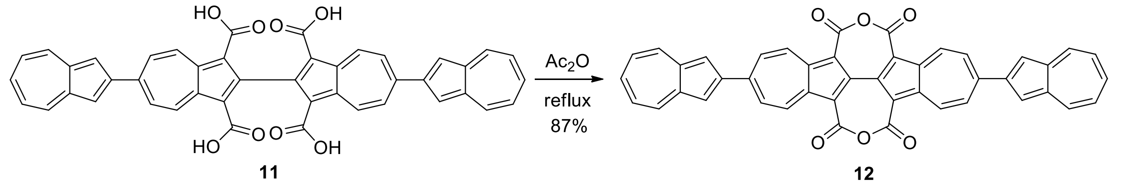

2,6':2',2'':6'',2'''-quaterazulene-1',1'',3',3''-tetracarboxylic dianhydride 12: A mixture of 2,6':2',2'':6'',2'''-quaterazulene-1',1'',3',3''-tetracarboxylic acid (205 mg, 0.3 mmol) and acetic anhydride (5 mL) was refluxed for 2 h. Then the mixture was filtrated to give product as a brown solid (169 mg, 87% yield). FT-IR (KBr, cm^{-1}) ν 2970.7, 1690.0, 1566.1, 1433.2, 1405.0, 1366.7, 1329.4, 1250.9, 1214.2, 1189.4, 1122.0, 993.6, 913.2, 895.2, 859.5, 810.2, 756.5, 691.2, 571.9, 532.4. MS (MALDI) m/z: 647.2 (M+H)$^+$.

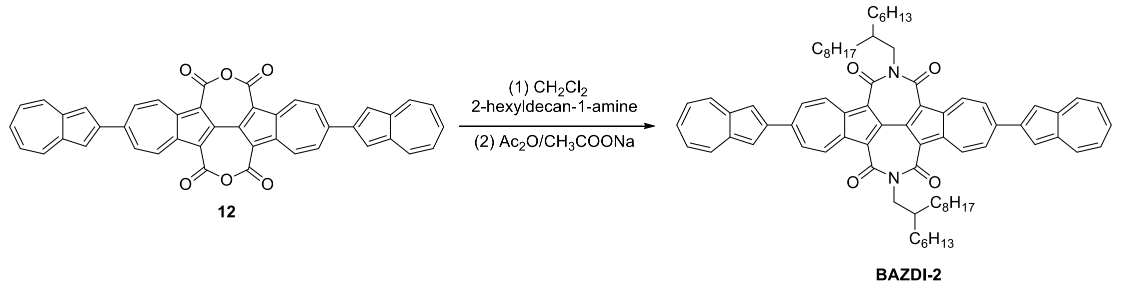

***N,N'*-bis(2-hexyldecyl)-2,6':2',2'':6'',2'''-quaterazulene-1',1'',3',3''-tetracarboxdiimide BAzDI-2:** A solution of 2-hexyldecan-1-amine solution (73 mg, 0.3 mmol) in 5 mL of dichloromethane was added dropwise to the solution of 2,6':2',2'':6'',2'''-quaterazulene-1',1'',3',3''-tetracarboxylic dianhydride **12** (65 mg, 0.1 mmol) in 10 mL of dichloromethane. The reaction was stirred under reflux for 4h. Upon removal of solvent, the residue was then added 10 mL of acetic anhydride and CH_3COONa (82 mg, 1.0 mmol). The resulting mixture was heated to reflux for another 4 h. The reaction mixture was diluted with water and thoroughly extracted with dichloromethane. The combined organic phases was washed with water and dried over Na_2SO_4. After concentrated under reduced pressure, the residue was purified by column chromatography on silica gel with dichloromethane/ hexane (1:2) to give product as red crystals (46 mg, 42% yield). M.p. = 282 °C. ^{1}H NMR (400 MHz, $CDCl_3$) δ (ppm): 9.63 (d, *J* = 11.2 Hz, 4H), 8.30 (d, *J* = 11.2 Hz, 4H), 8.27 (d, *J* = 9.3 Hz, 4H), 7.68 (s, 4H), 7.50 (t, *J* = 9.9 Hz, 2H), 7.12 (dd, *J* = 9.9 Hz, 9.3 Hz, 4H), 4.45

(d, J = 7.5 Hz, 4H), 2.10 (s, 2H), 1.29 (m, 48H), 0.79 (t, J = 6.7 Hz, 12H). ^{13}C NMR (100 MHz, $CDCl_3$) δ 165.7, 149.4, 149.3, 142.2, 140.9, 138.6, 138.3, 137.9, 135.8, 130.3, 124.0, 118.2, 116.0, 49.7, 36.9, 31.4, 29. 8, 29.4, 29.2, 29.0, 26.0, 22.2, 22.2, 13.6. FT-IR (KBr, cm^{-1}) ν 2921.1, 2850.3, 2363.1, 1646.2, 1613.4, 1567.8, 1479.6, 1425.5, 1386.8, 1329.2, 1256.1, 1171.5, 1017.8, 943.4, 915.6, 895.9, 853.9, 803.4, 779.8, 759.8, 724.2, 681.5, 607.8, 573.9, 533.2, 408.5. Anal. Calcd for $C_{76}H_{88}O_4N_2$: C, 83.47; H, 8.11; N, 2.56. Found: C, 83.61; H, 8.03; N, 2.39. MS (MALDI) m/z: 1093.7 $(M+H)^+$. HRMS (MALDI-FT) (m/z): $(M+H)^+$ Calcd for $C_{76}H_{89}O_4N_2$ 1093.6817; Found, 1093.6812.

3. TGA and DSC curves for BAzDI-1 and BAzDI-2.

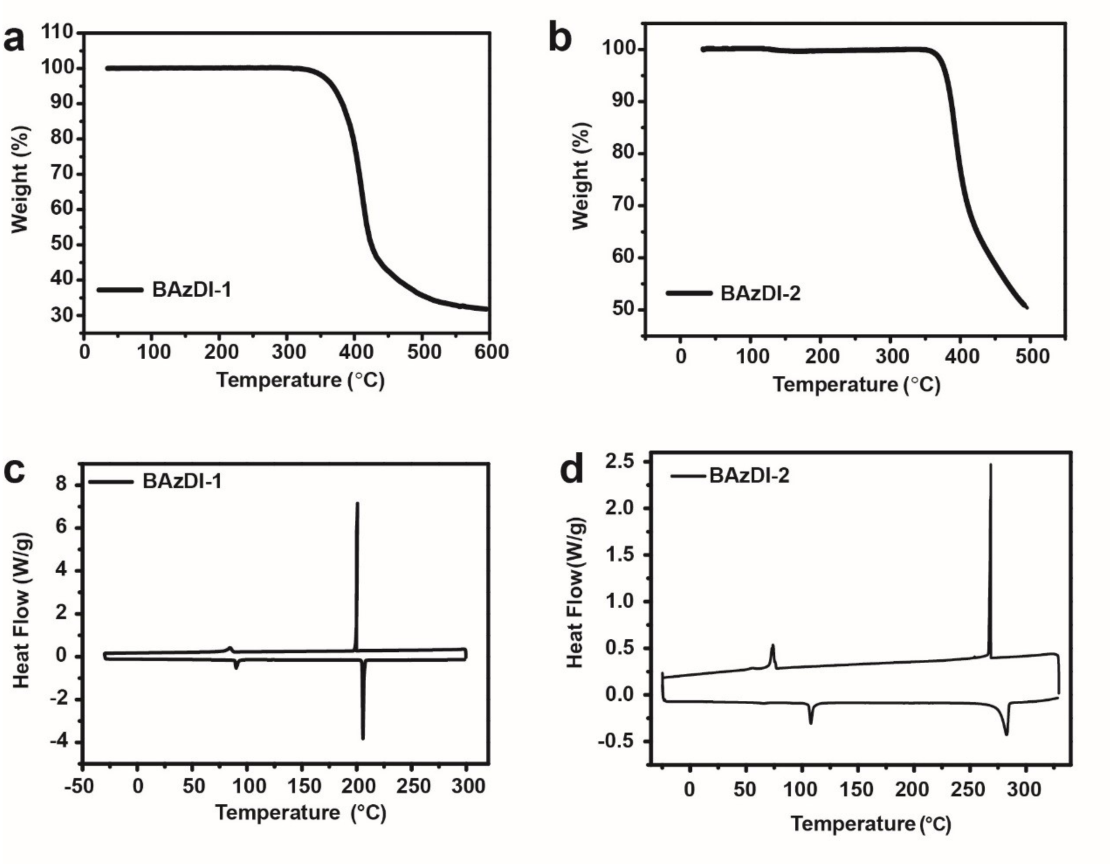

Figure S1. (a) TGA measurements for **BAzDI-1**. (b) TGA measurements for **BAzDI-2**. (c) DSC measurements for **BAzDI-1**. (d) DSC measurements for **BAzDI-2**.

4. X-ray Crystallographic Structure for BAzDI-1.

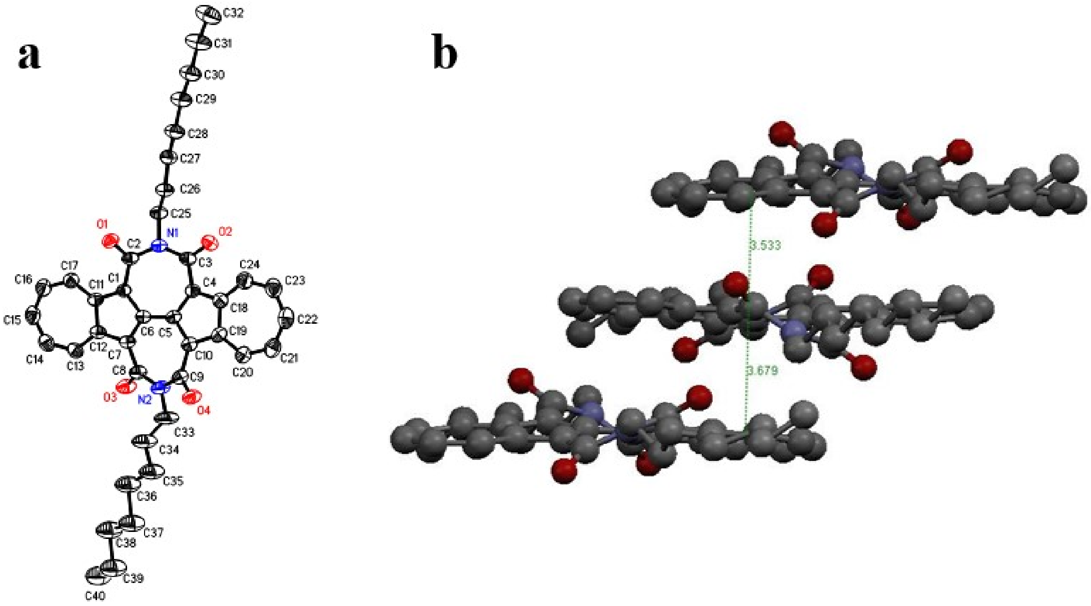

Figure S2. (a) ORTEP diagram of **BAzDI-1**. (b) Interplannar distance of **BAzDI-1**.

Table S1. Crystal data and structure refinement for **BAzDI-1**.

Empirical formula	C40 H44 N2 O4	
Formula weight	616.77	
Temperature	293(2) K	
Wavelength	0.71073 Å	
Crystal system	Triclinic	
Space group	P -1	
Unit cell dimensions	a = 10.124(2) Å	a= 90.017(5)°.
	b = 10.455(3) Å	b= 94.225(7)°.
	c = 17.030(4) Å	g = 109.592(6)°.
Volume	1693.0(7) $Å^3$	
Z	2	
Density (calculated)	1.210 Mg/m^3	
Absorption coefficient	0.078 mm^{-1}	
F(000)	660	
Crystal size	0.180 x 0.110 x 0.040 mm^3	
Theta range for data collection	2.068 to 25.498°.	
Index ranges	-11<=h<=12, -12<=k<=12, -16<=l<=20	
Reflections collected	9989	
Independent reflections	6310 [R(int) = 0.0476]	
Completeness to theta = 25.242°	99.9 %	
Absorption correction	Semi-empirical from equivalents	
Max. and min. transmission	0.7456 and 0.5895	
Refinement method	Full-matrix least-squares on F^2	
Data / restraints / parameters	6310 / 98 / 491	
Goodness-of-fit on F^2	0.990	
Final R indices [I>2sigma(I)]	R1 = 0.0785, wR2 = 0.1806	
R indices (all data)	R1 = 0.1956, wR2 = 0.2416	
Largest diff. peak and hole	0.247 and -0.214 e.$Å^{-3}$	

5. The geometries of 2,2′-biazulene, BAzDI-1 and BAzDI-2 obtained by DFT calculations.

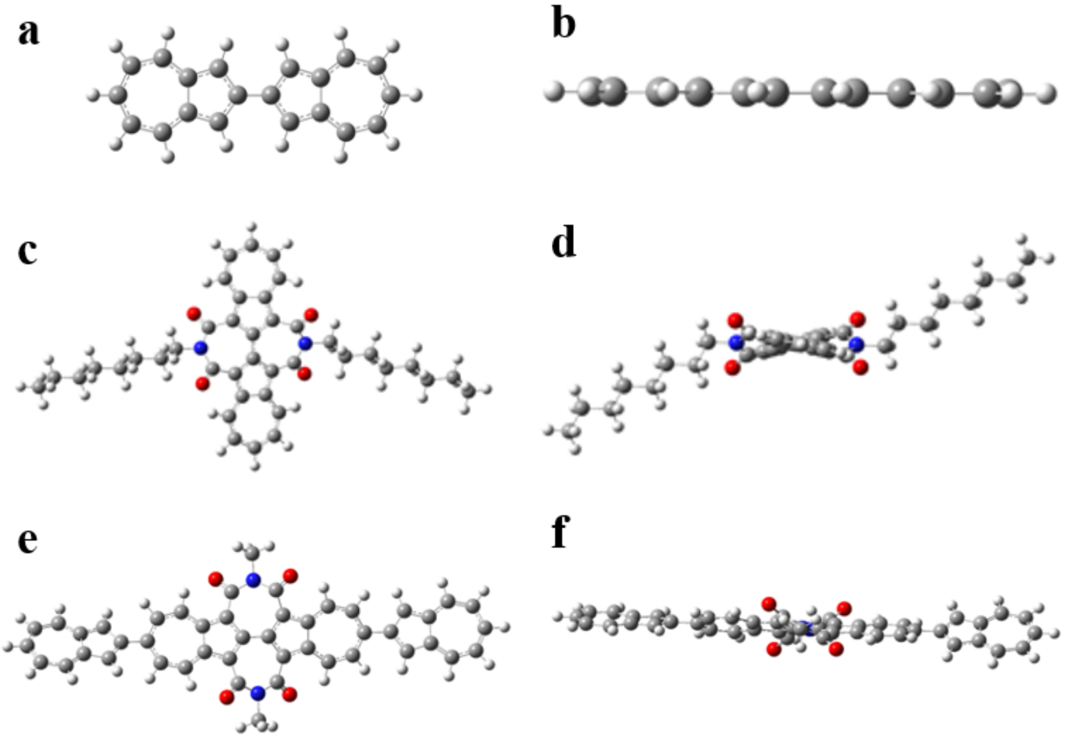

Figure S3. The geometries of 2,2′-biazulene (a, b), **BAzDI-1** (c, d), and N,N'-bis(methyl)-substituted model molecule for **BAzDI-2** (e, f), obtained by DFT calculations.

6. UV-Vis spectra of BAzDI-1 and BAzDI-2 in solution and thin film.

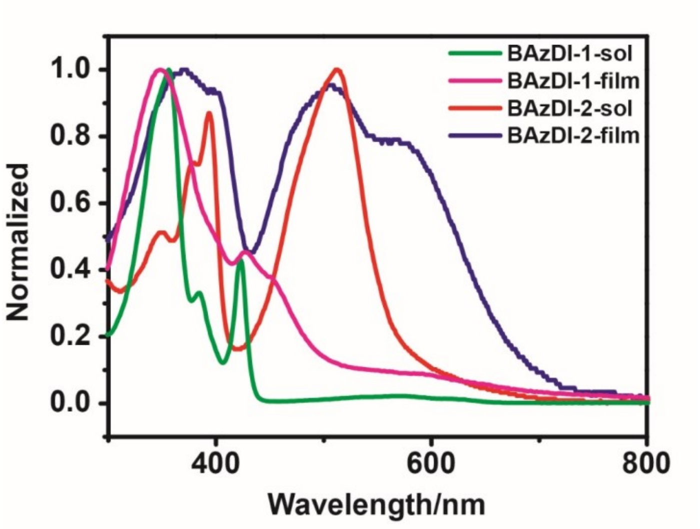

Figure S4. UV-vis spectra of **BAzDI-1** (green, in CH_2Cl_2; pink, as-spun film) and **BAzDI-2** (red, in CH_2Cl_2; blue, as-spun film).

7. Spectra and color of BAzDI-1 and PDI.

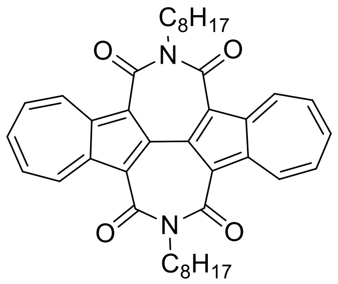

BAzDI-1

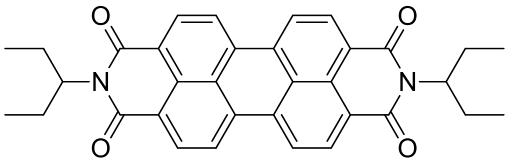

PDI

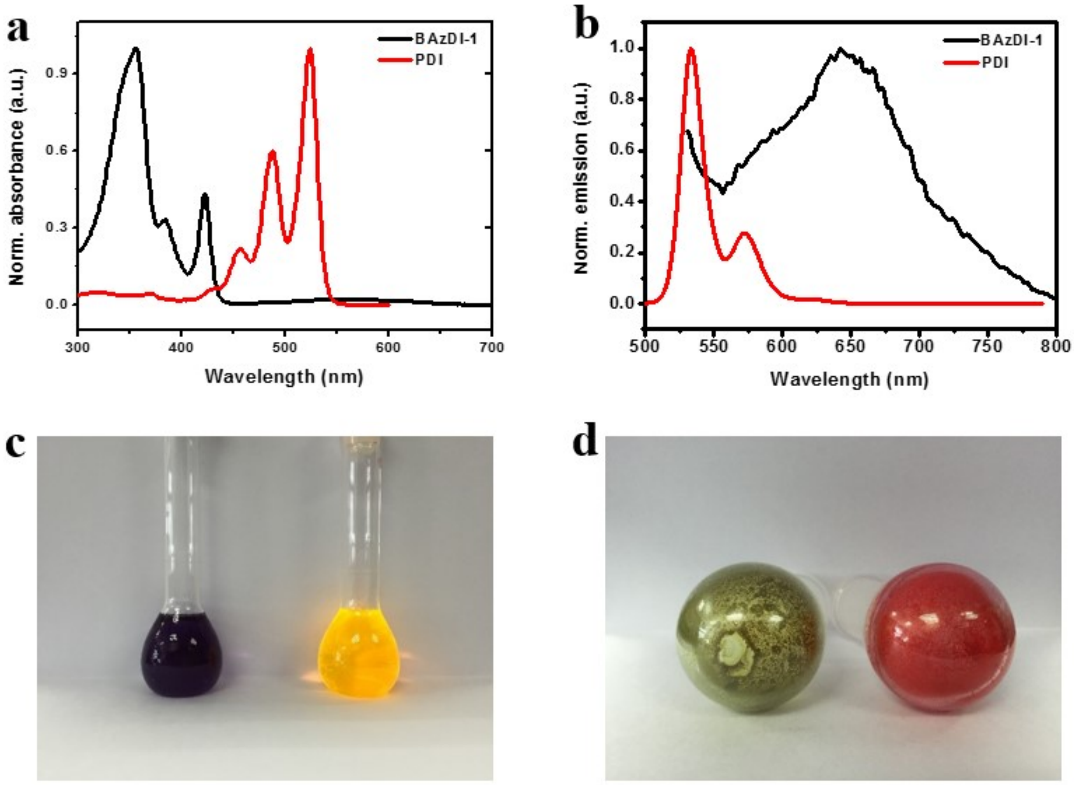

Figure S5. (a) Absorption spectra of **BAzDI-1** and N,N'-bis(1-ethylpropyl)-3,4:9,10-perylenebis(dicarboximide) **PDI**. (b) Emission spectra of **BAzDI-1** (no fluorescence was observed at room temperature, and only very weak fluorescence was measured at –198 °C) and **PDI** (very strong fluorescence, measured at room temperature). (c) Color of **BAzDI-1** (violet, in CH_2Cl_2) and **PDI** (yellow, in CH_2Cl_2). (d) Color of **BAzDI-1** (green, solid state) and **PDI** (red, solid state).

8. Characteristics of OFET devices.

Table S2. Characteristics of OFETs Based on **BAzDI-2** at Different Annealing Temperatures.

Annealing Temperature(°C)	μ_e (μ_{ave}) $cm^2 V^{-1} s^{-1}$	I_{On}/I_{Off}	V_T (V)
RT	3.0×10^{-4} (2.3×10^{-4})	10^4~10^5	38 – 58
80 °C	1.2×10^{-2} (0.8×10^{-2})	10^4~10^5	42 – 56
120 °C	1.5×10^{-2} (1.3×10^{-2})	10^4~10^5	50 – 65

9. XRD and AFM measurements for BAzDI-2.

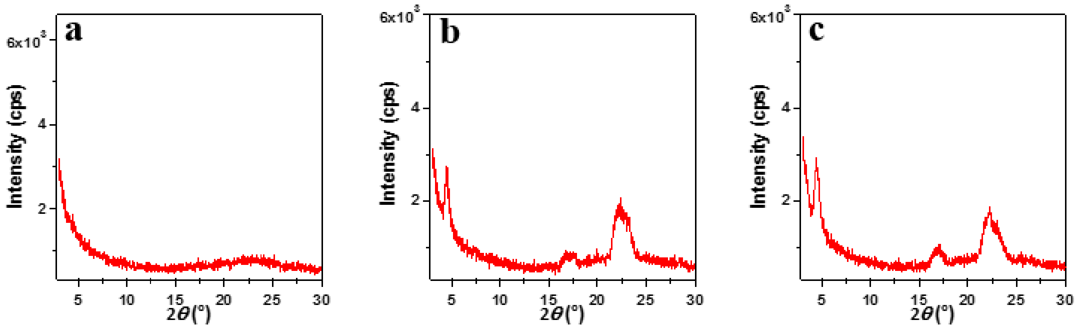

Figure S6. XRD patterns of spin-coated thin films of **BAzDI-2** annealed at room temperature (a), 80 °C (b) and 120 °C (c).

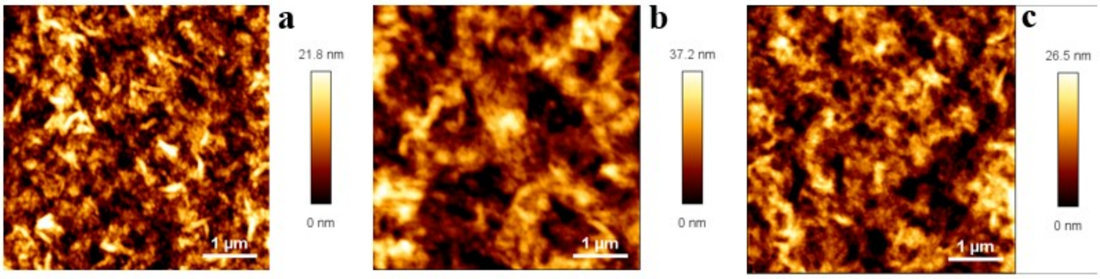

Figure S7. AFM images of spin-coated thin films of BAzDI-2 annealed at room temperature (a), 80 °C (b) and 120 °C.

10. NMR, MS and IR spectra.

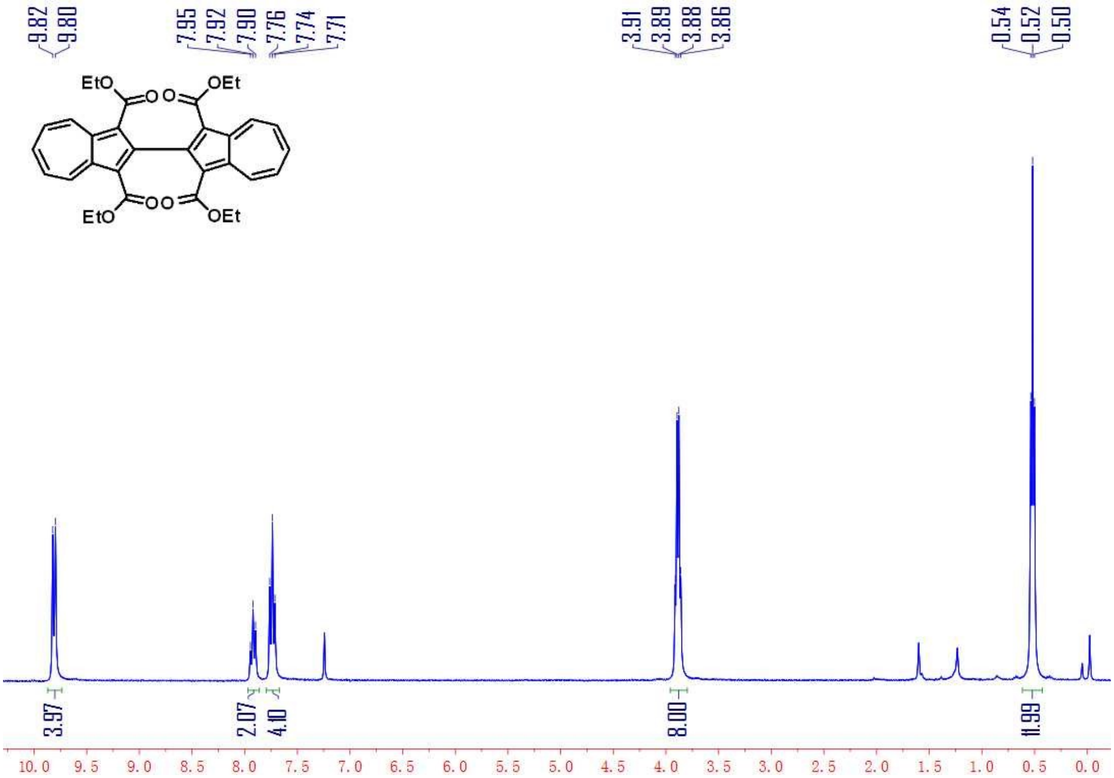

Figure S8. ^{1}H NMR spectrum of **2** (400 MHz, $CDCl_3$).

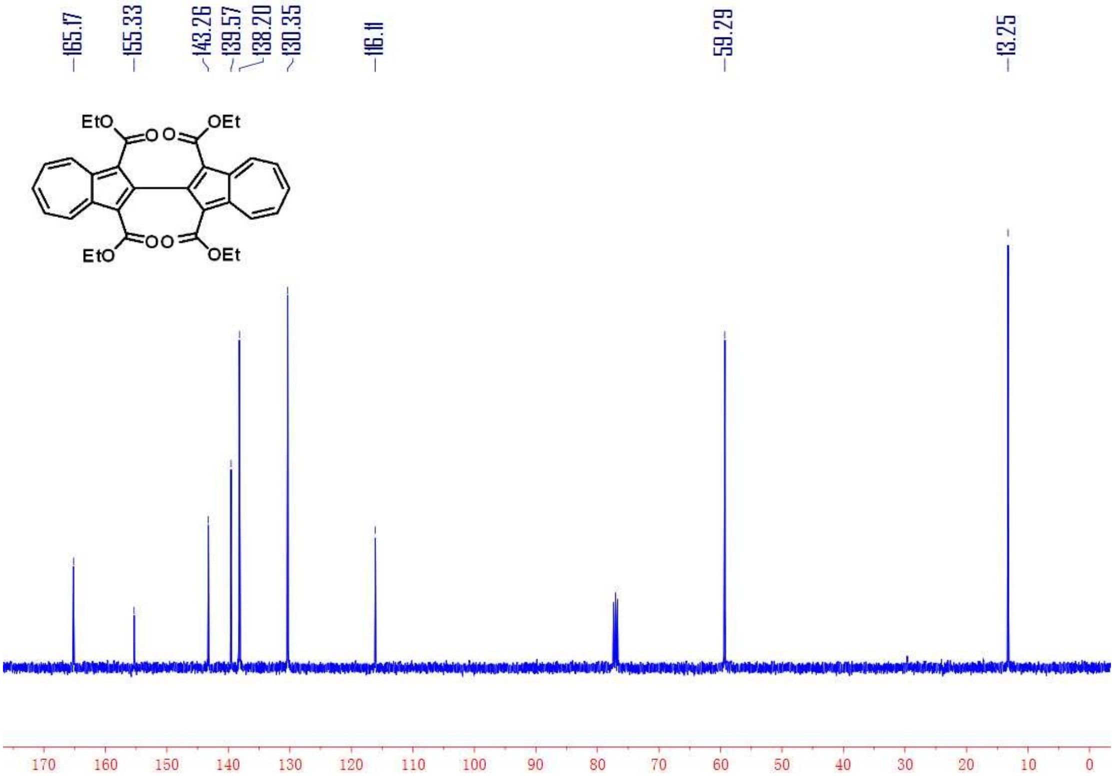

Figure S9. ^{13}C NMR spectrum of **2** (100 MHz, $CDCl_3$).

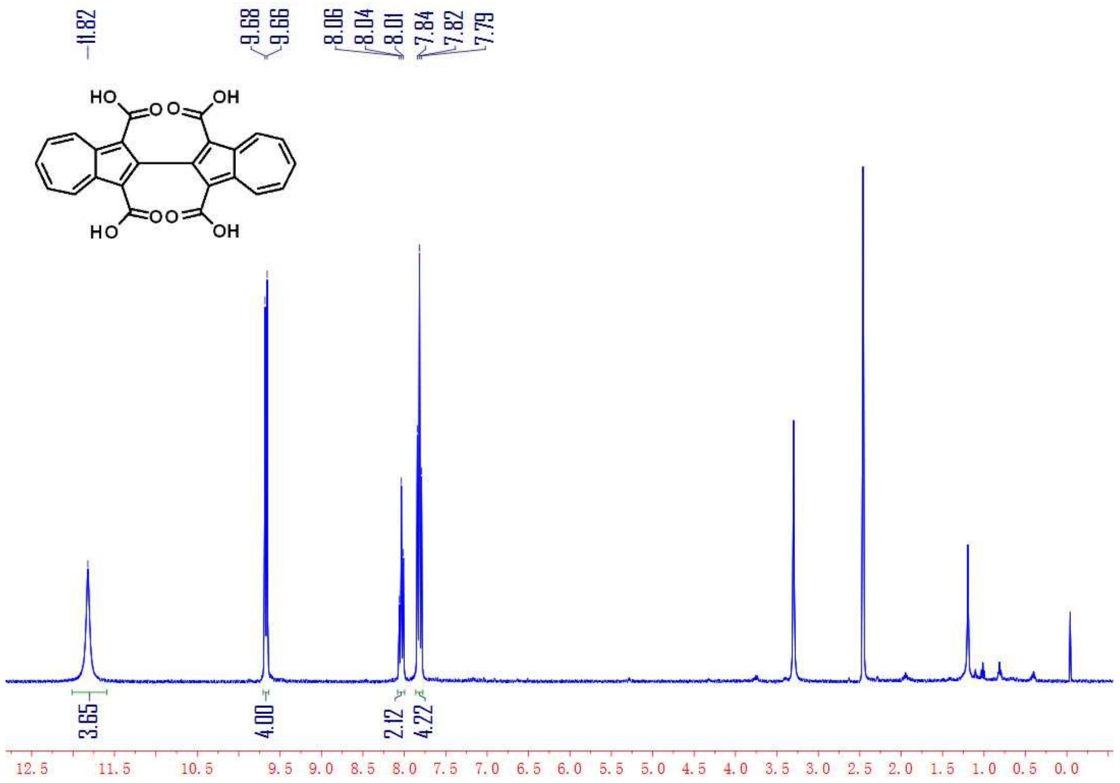

Figure S10. ^{1}H NMR spectrum of **3** (400 MHz, DMSO-d_6).

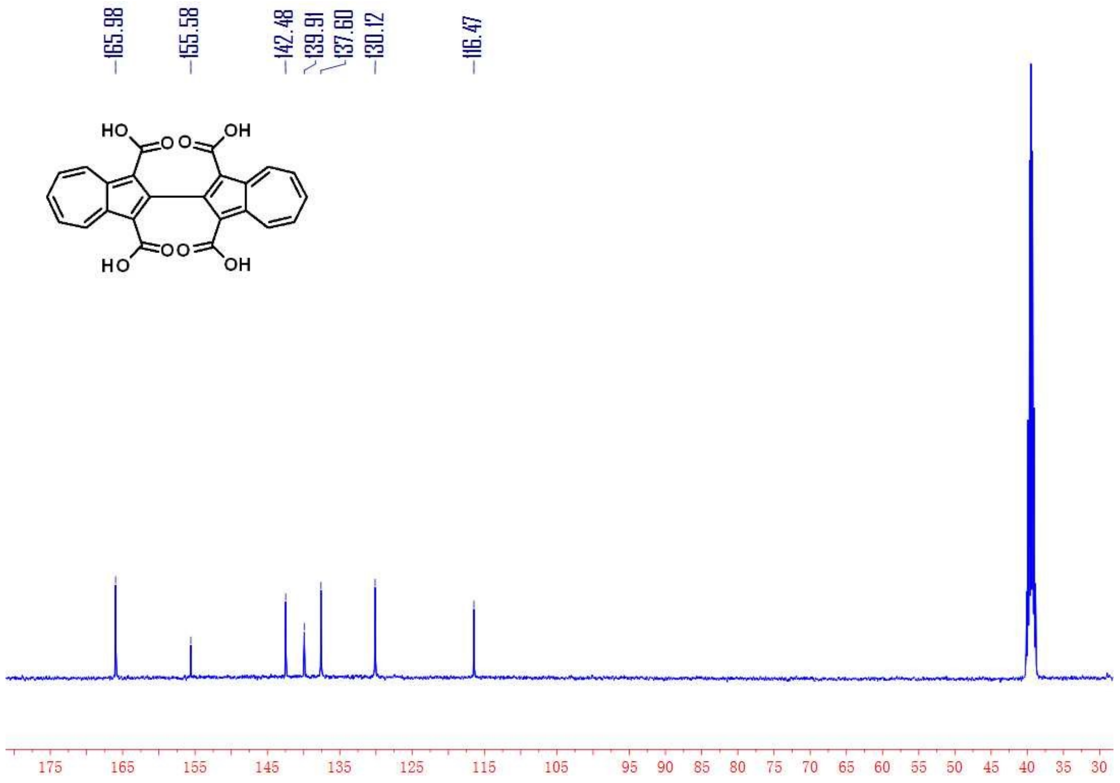

Figure S11. ^{13}C NMR spectrum of **3** (100 MHz, DMSO-d_6).

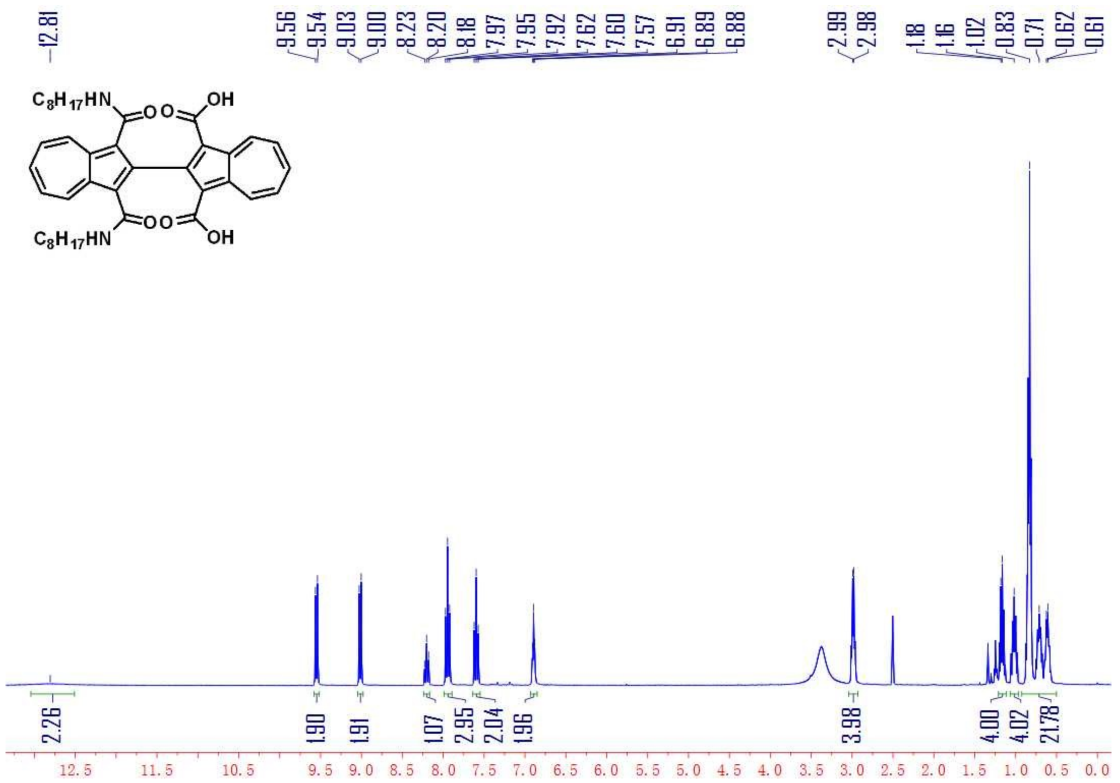

Figure S12. ^{1}H NMR spectrum of **5** (400 MHz, DMSO-d_6).

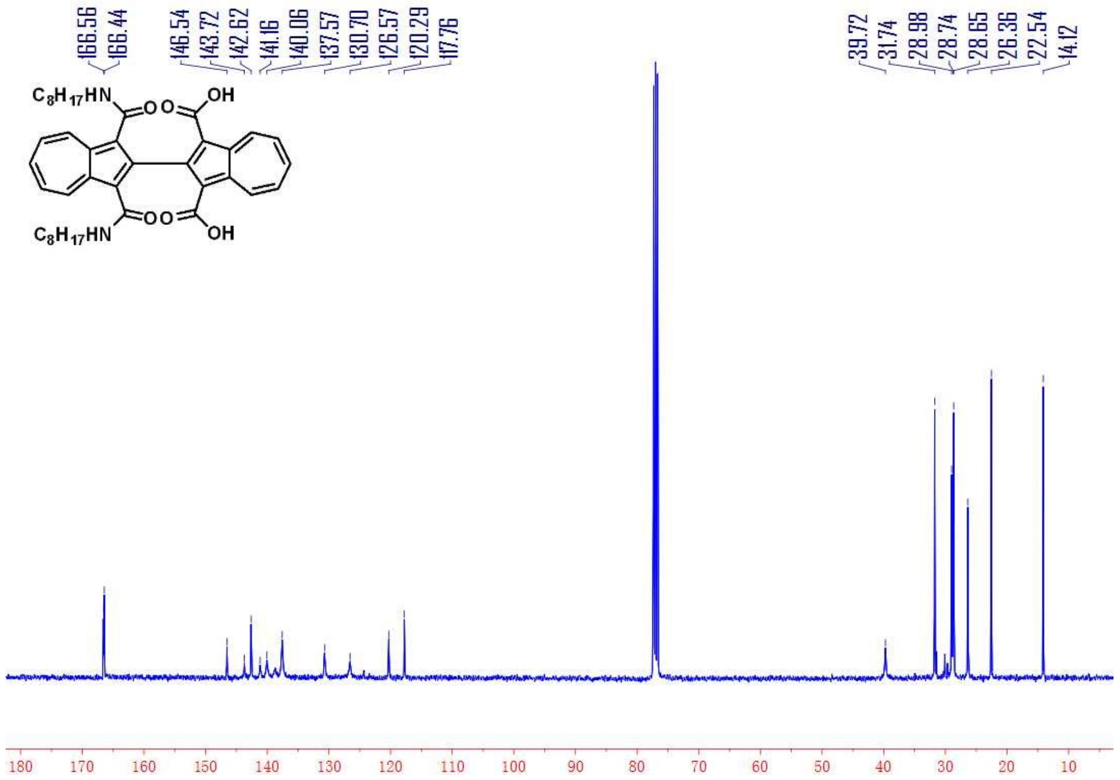

Figure S13. ^{13}C NMR spectrum of **5** (100 MHz, $CDCl_3$).

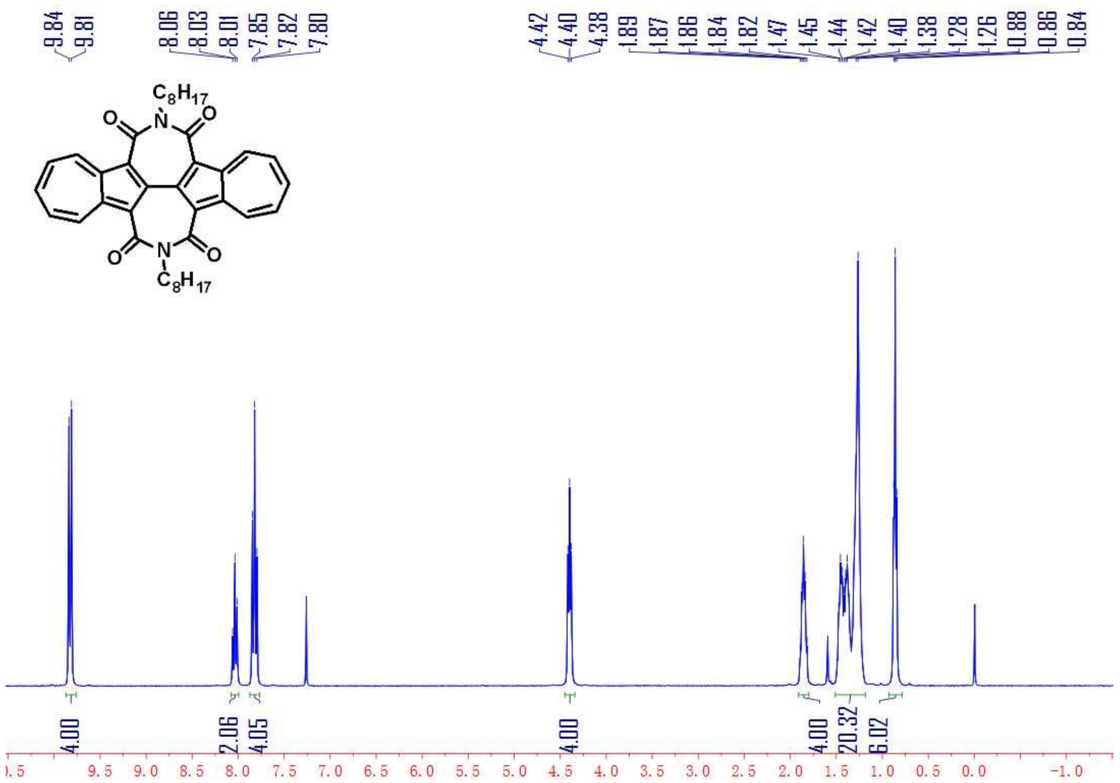

Figure S14. ^{1}H NMR spectrum of **BAzDI-1** (400 MHz, $CDCl_3$).

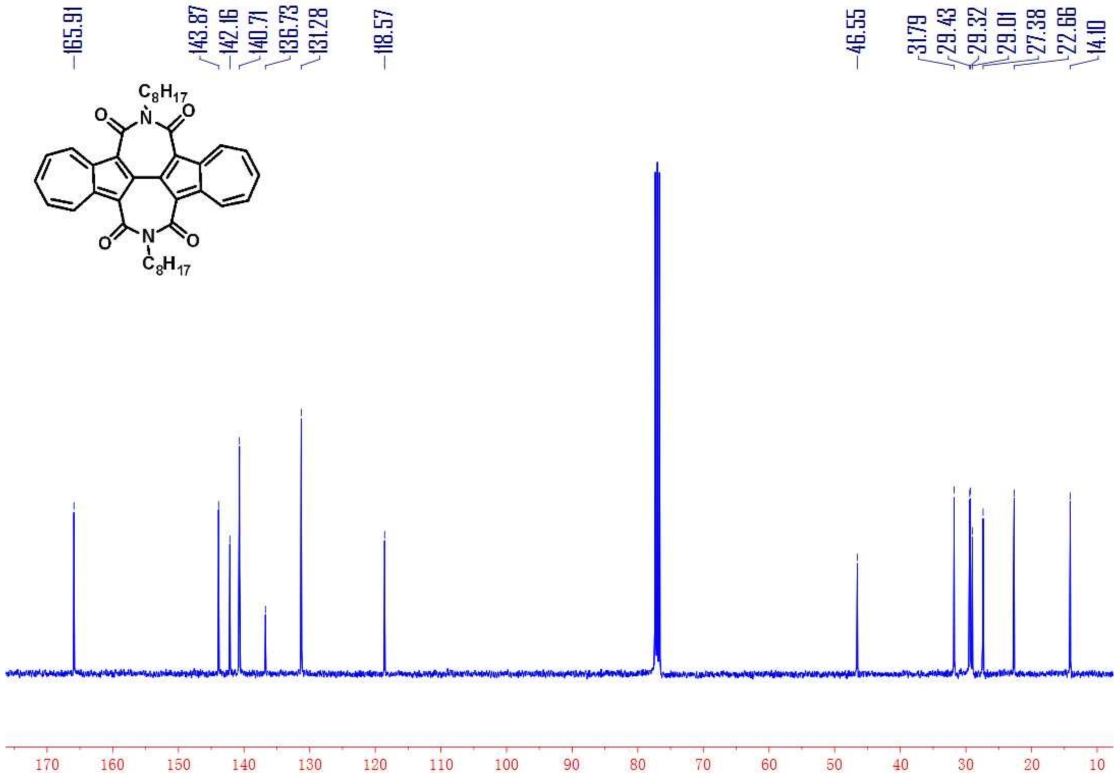

Figure S15. ^{13}C NMR spectrum of **BAzDI-1** (100 MHz, $CDCl_3$).

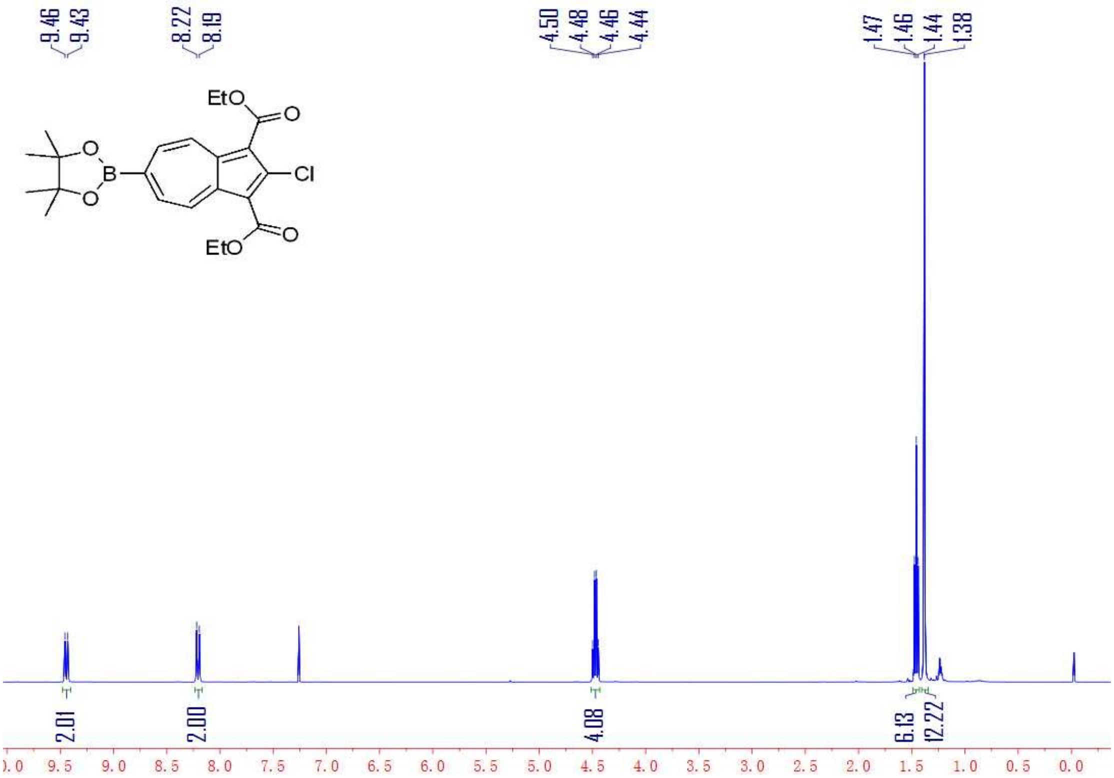

Figure S16. ^{1}H NMR spectrum of **7** (400 MHz, $CDCl_3$).

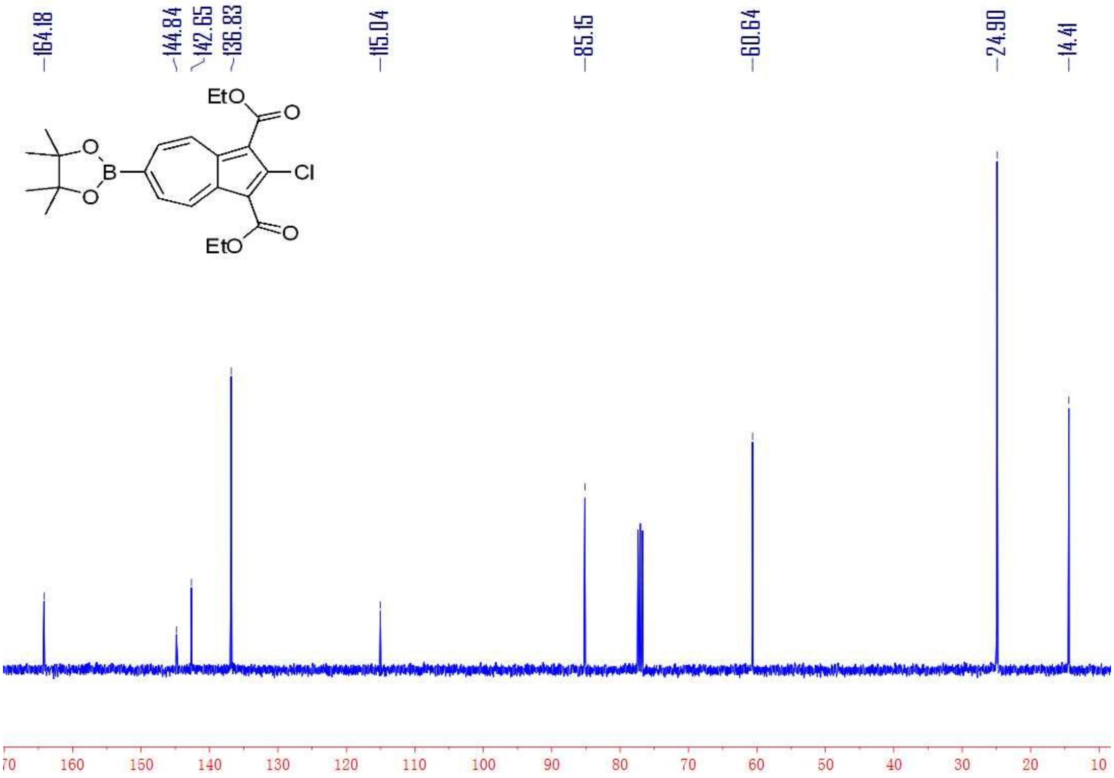

Figure S17. ^{13}C NMR spectrum of **7** (100 MHz, $CDCl_3$).

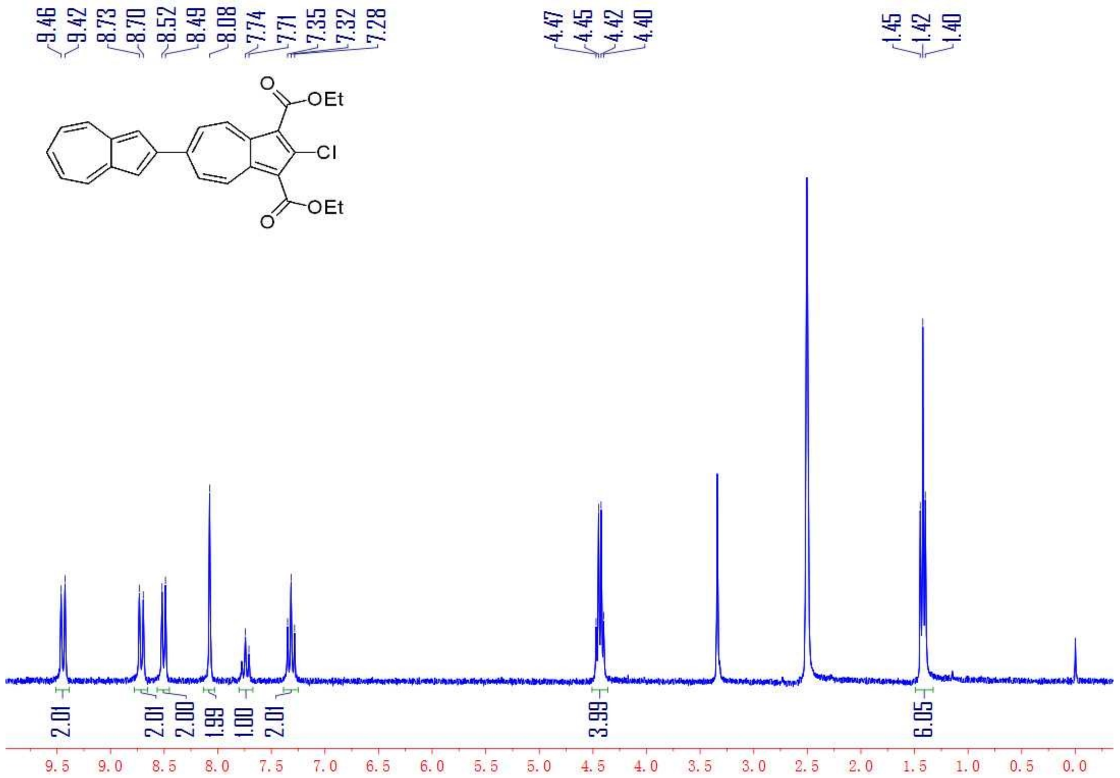

Figure S18. ^{1}H NMR spectrum of **9** (300 MHz, DMSO-d_6).

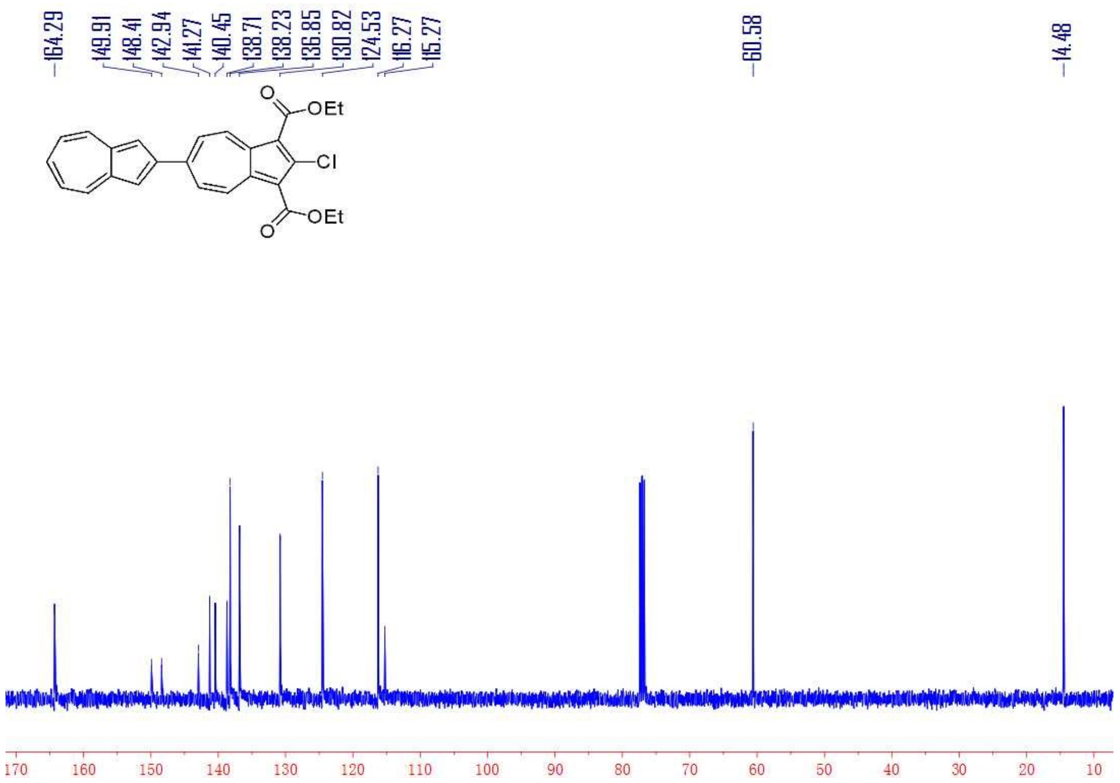

Figure S19. ^{13}C NMR spectrum of **9** (100 MHz, $CDCl_3$).

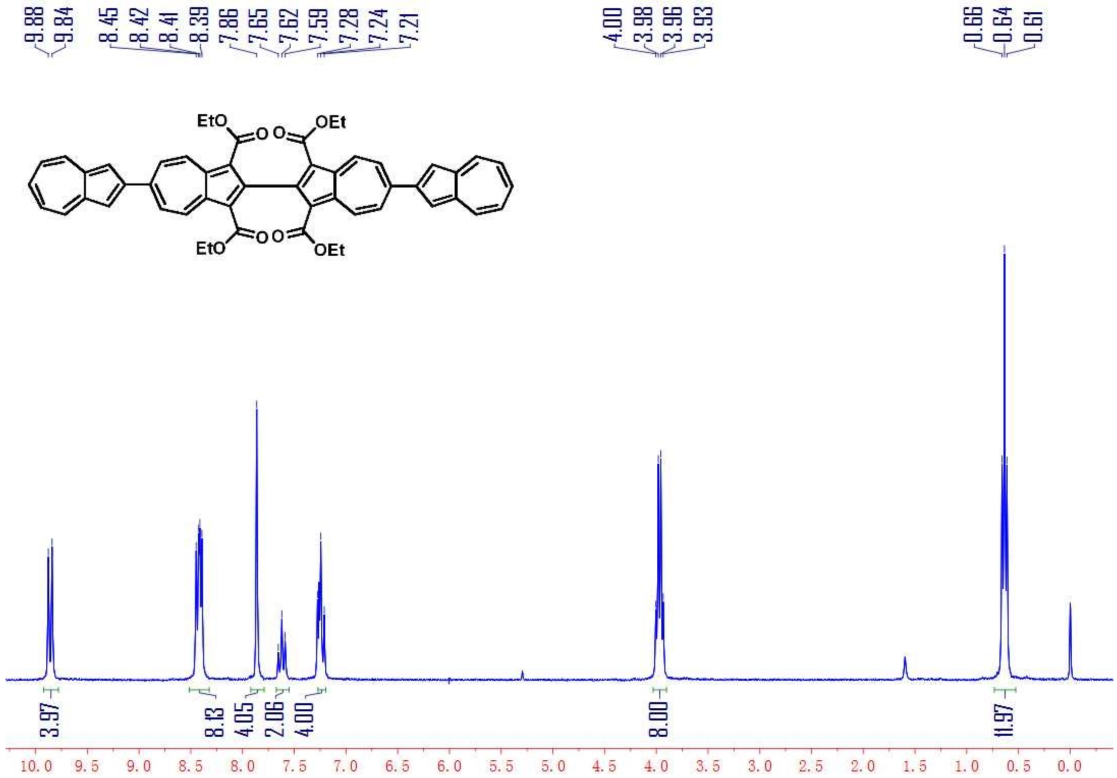

Figure S20. ^{1}H NMR spectrum of **10** (300 MHz, $CDCl_3$).

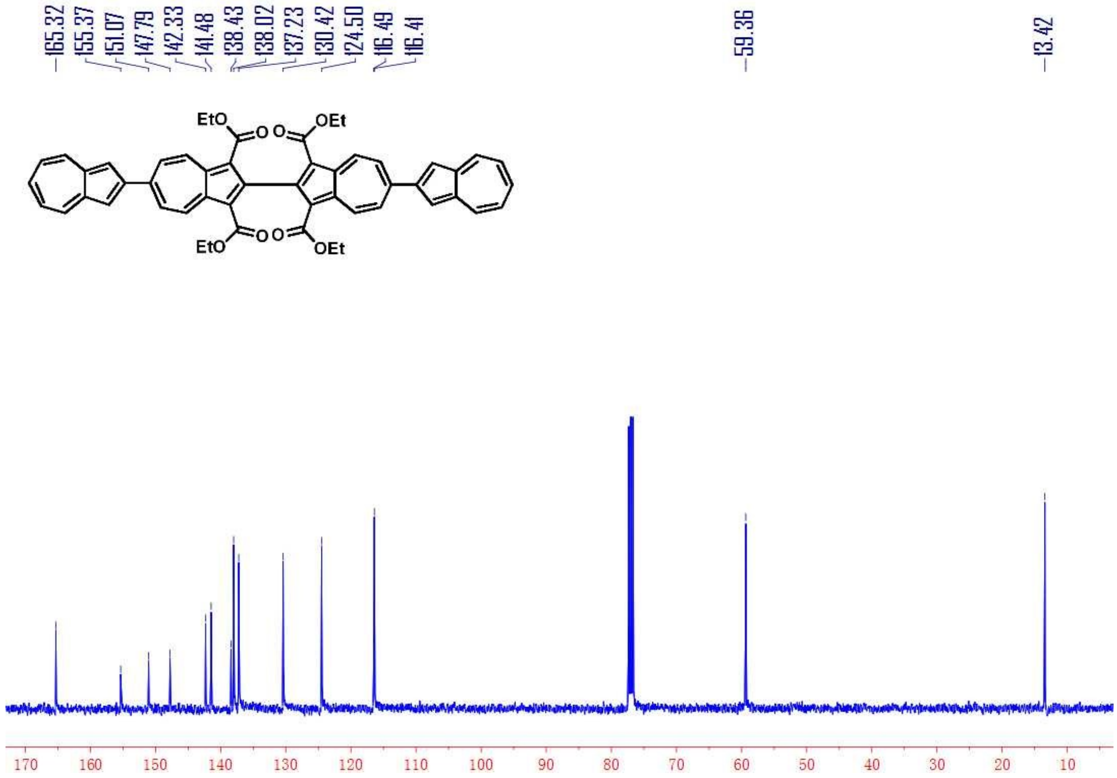

Figure S21. ^{13}C NMR spectrum of **10** (100 MHz, $CDCl_3$).

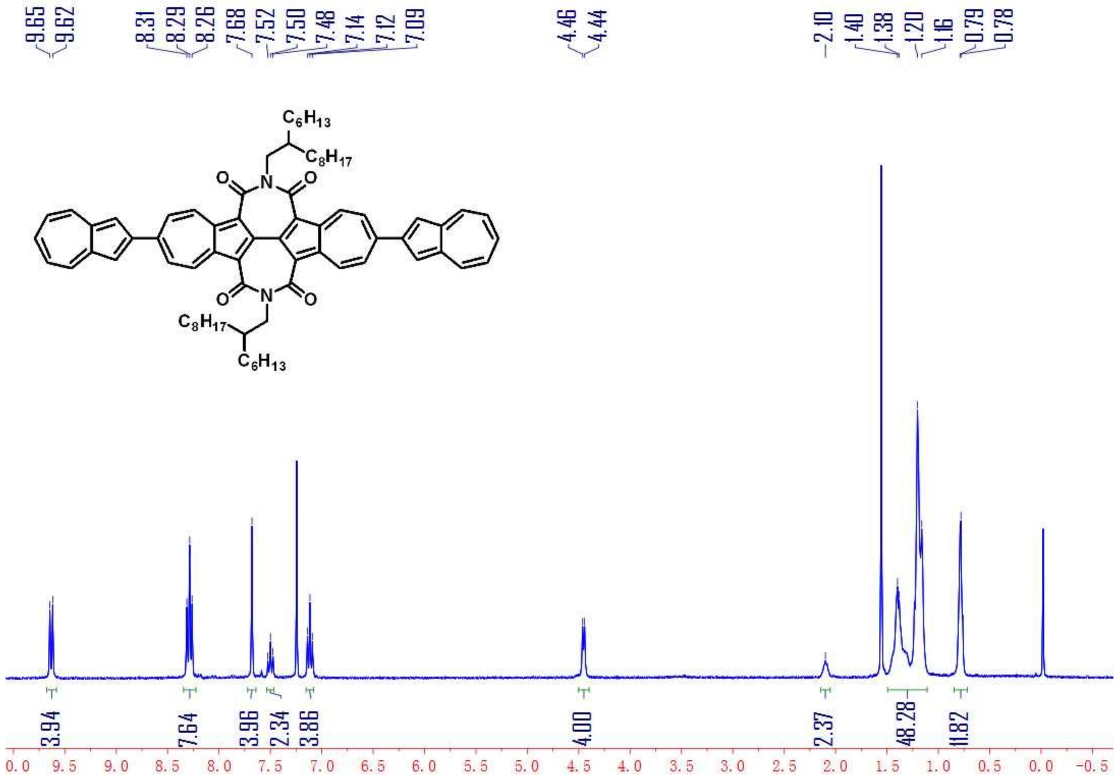

Figure S22. ^{1}H NMR spectrum of **BAzDI-2** (400 MHz, $CDCl_3$).

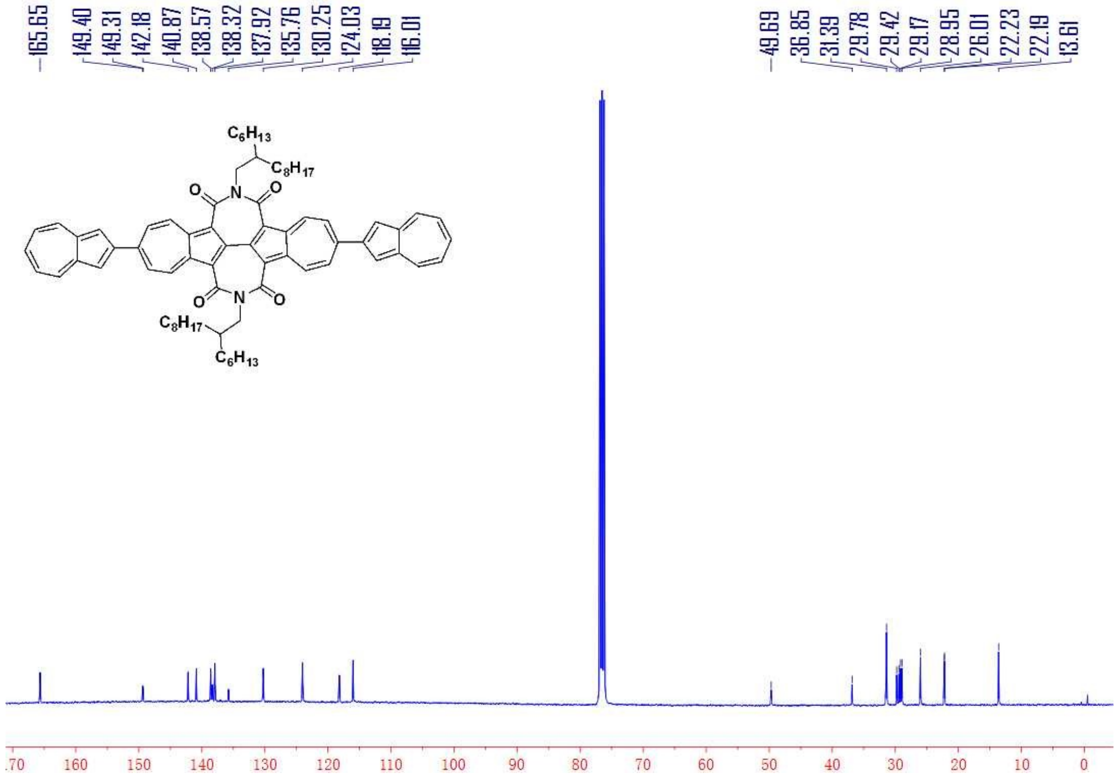

Figure S23. ^{13}C NMR spectrum of **BAzDI-2** (100 MHz, $CDCl_3$)

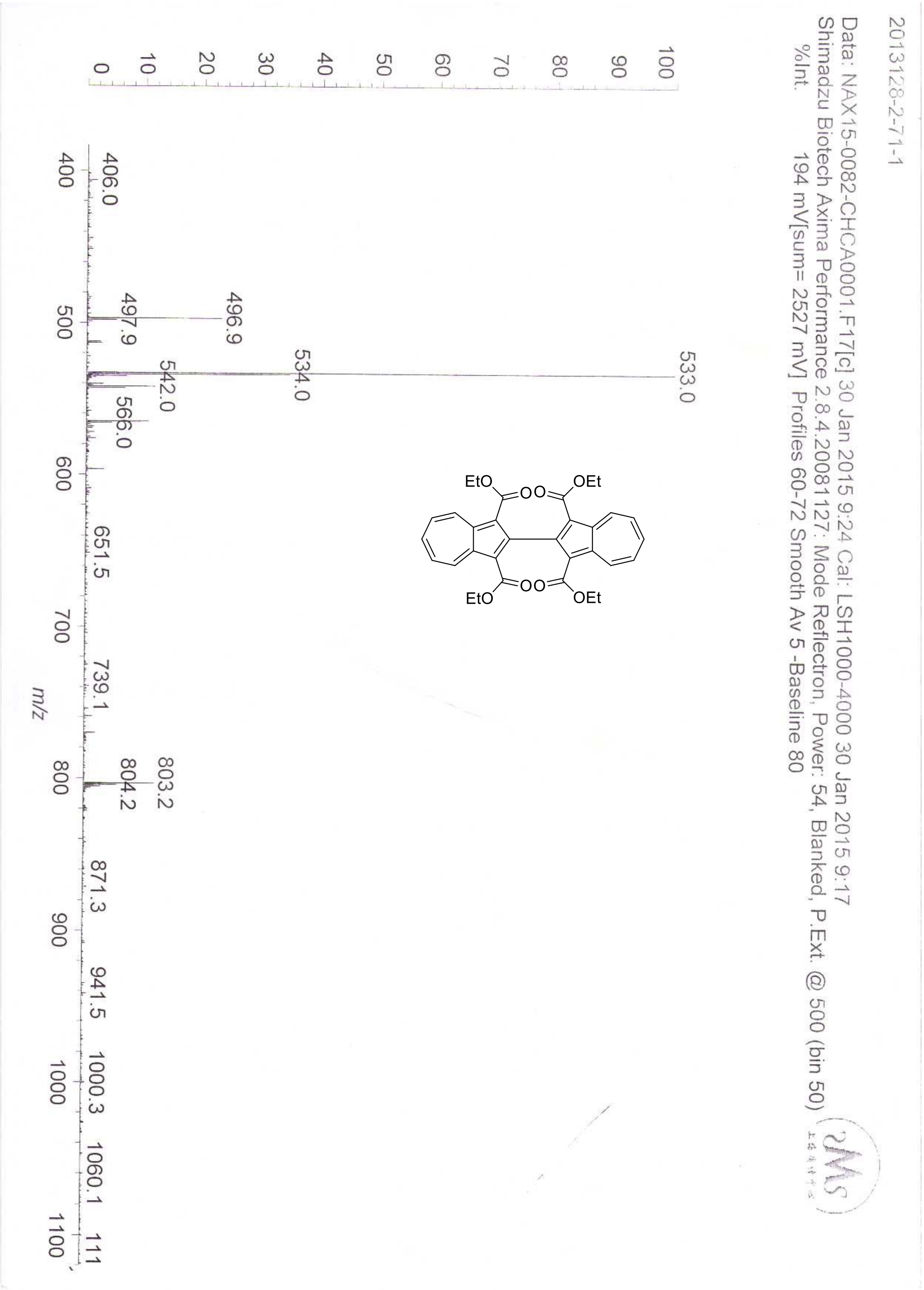

Figure S24. MS spectrum of **2**.

National Center for Organic Mass Spectrometry in Shanghai
Shanghai Institute of Organic Chemistry
Chinese Academic of Sciences
High Resolution MS DATA REPORT

Instrument: Thermo Fisher Scientific LTQ FT Ultra

Card Serial Number : E150477

Sample Serial Number: 2-71-1

Operator : HUAQIN Date: 2015/02/05

Operation Mode: DART Postive

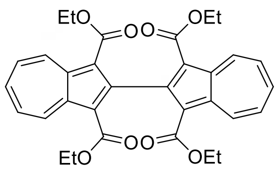

Elemental composition search on mass 543.20

m/z= 538.20-548.20

m/z	Theo. Mass	Delta (ppm)	RDB equiv.	Composition
543.2000	543.2000	0.03	18.0	$C_{30}H_{29}O_7N_3$
	543.2000	0.04	23.5	$C_{29}H_{23}O_2N_{10}$
	543.2005	-0.90	5.5	$C_{16}H_{31}O_{13}N_8$
	543.2013	-2.43	23.0	$C_{31}H_{25}O_3N_7$
	543.2013	-2.44	17.5	$C_{32}H_{31}O_8$
	543.1987	2.51	18.5	$C_{28}H_{27}O_6N_6$
	543.2019	-3.37	5.0	$C_{18}H_{33}O_{14}N_5$
	543.2027	-4.90	22.5	$C_{33}H_{27}O_4N_4$
	543.1973	4.97	13.5	$C_{27}H_{31}O_{10}N_2$
	543.1973	4.98	19.0	$C_{26}H_{25}O_5N_9$

Figure S25. HRMS spectrum of **2**.

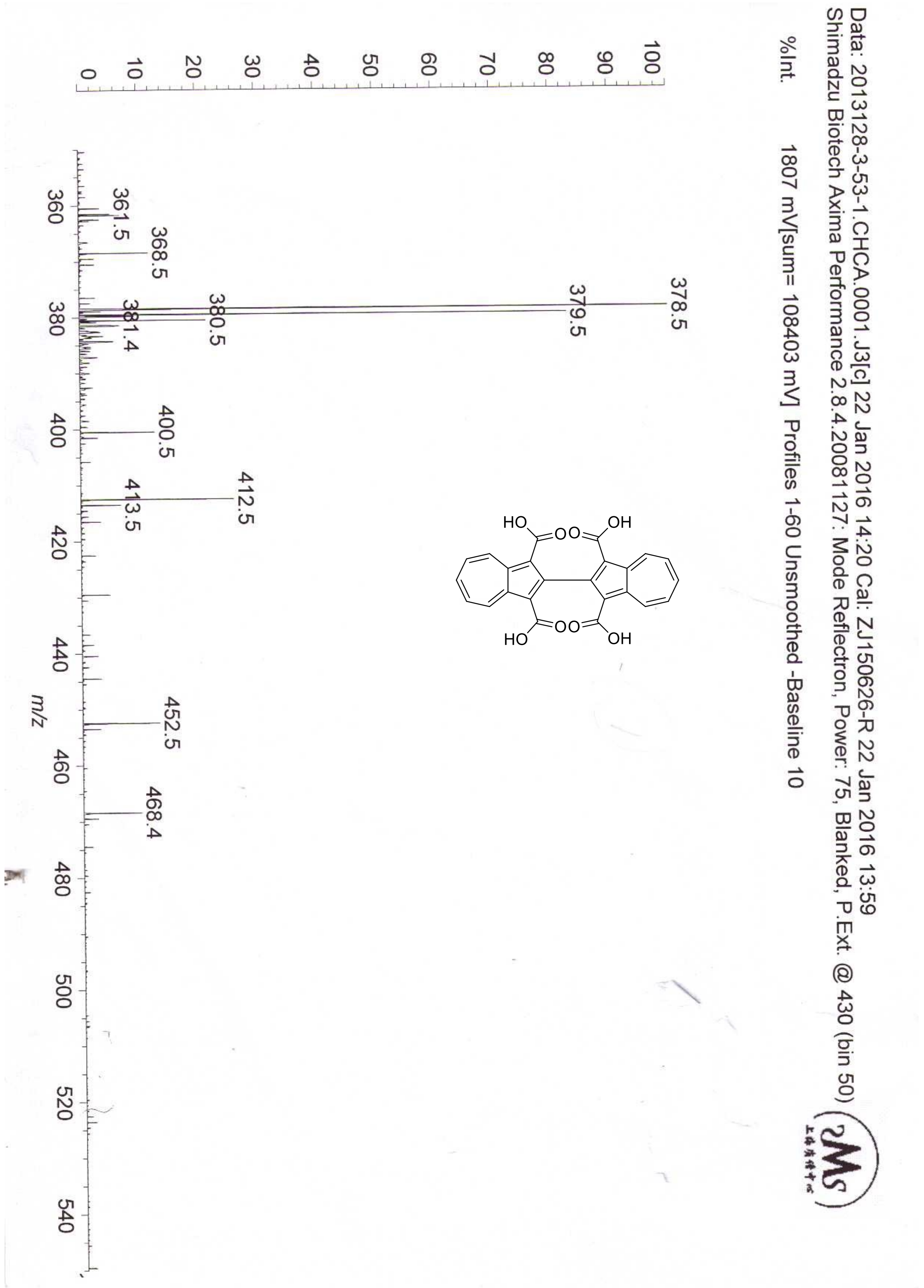

Figure S26. MS spectrum of **3**

National Center for Organic Mass Spectrometry in Shanghai
Shanghai Institute of Organic Chemistry
Chinese Academic of Sciences
High Resolution MS DATA REPORT

Instrument: Thermo Fisher Scientific LTQ FT Ultra

Card Serial Number : E161253

Sample Serial Number: 2013128-3-53-1

Operator : ZHUFJ Date: 2016/05/05

Operation Mode: DART Positive

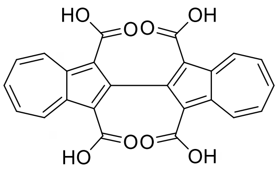

Elemental composition search on mass 453.06

m/z= 448.06-458.06

m/z	Theo. Mass	Delta (ppm)	RDB equiv.	Composition
453.0577	453.0581	-0.97	17.5	$C_{24} H_{14} O_8 Na$
	453.0565	2.60	16.5	$C_{21} H_{13} O_{10} N_2$

Figure S27. HRMS spectrum of **3**

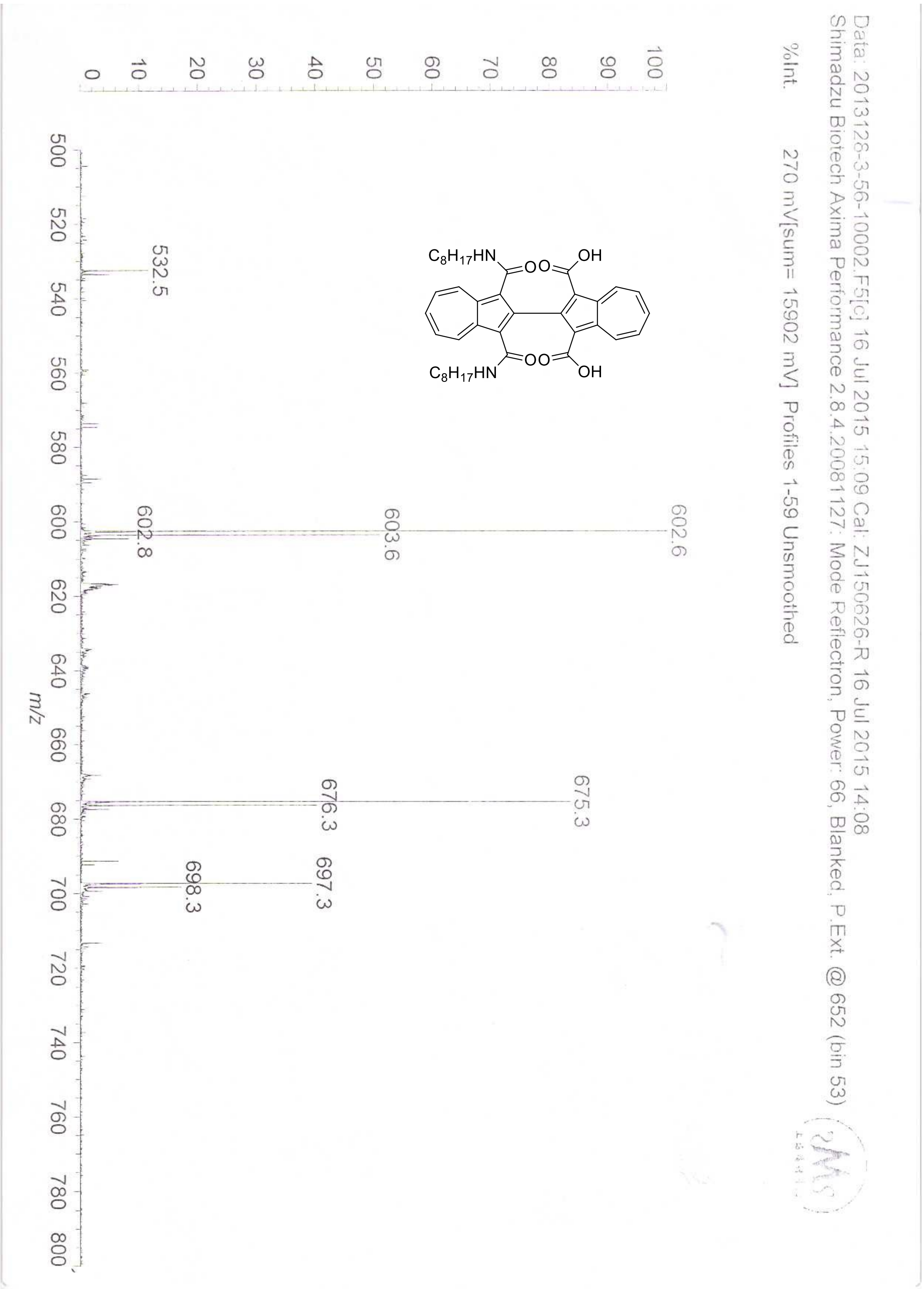

Figure S28. MS spectrum of **5**

National Center for Organic Mass Spectrometry in Shanghai
Shanghai Institute of Organic Chemistry
Chinese Academic of Sciences
High Resolution MS DATA REPORT

Instrument: Thermo Fisher Scientific LTQ FT Ultra

Card Serial Number : M152692

Sample Serial Number: 3-56-1

Operator : HUAQIN Date: 2015/09/29

Operation Mode: MALDI-FT_DHB

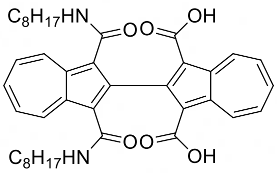

Elemental composition search on mass 653.36

m/z= 648.36-658.36

m/z	Theo. Mass	Delta (ppm)	RDB equiv.	Composition
653.3586	653.3585	0.09	17.5	$C_{40}H_{49}O_6N_2$
	653.3572	2.14	18.0	$C_{38}H_{47}O_5N_5$
	653.3612	-4.02	22.0	$C_{43}H_{47}O_3N_3$

Figure S29. HRMS spectrum of **5**

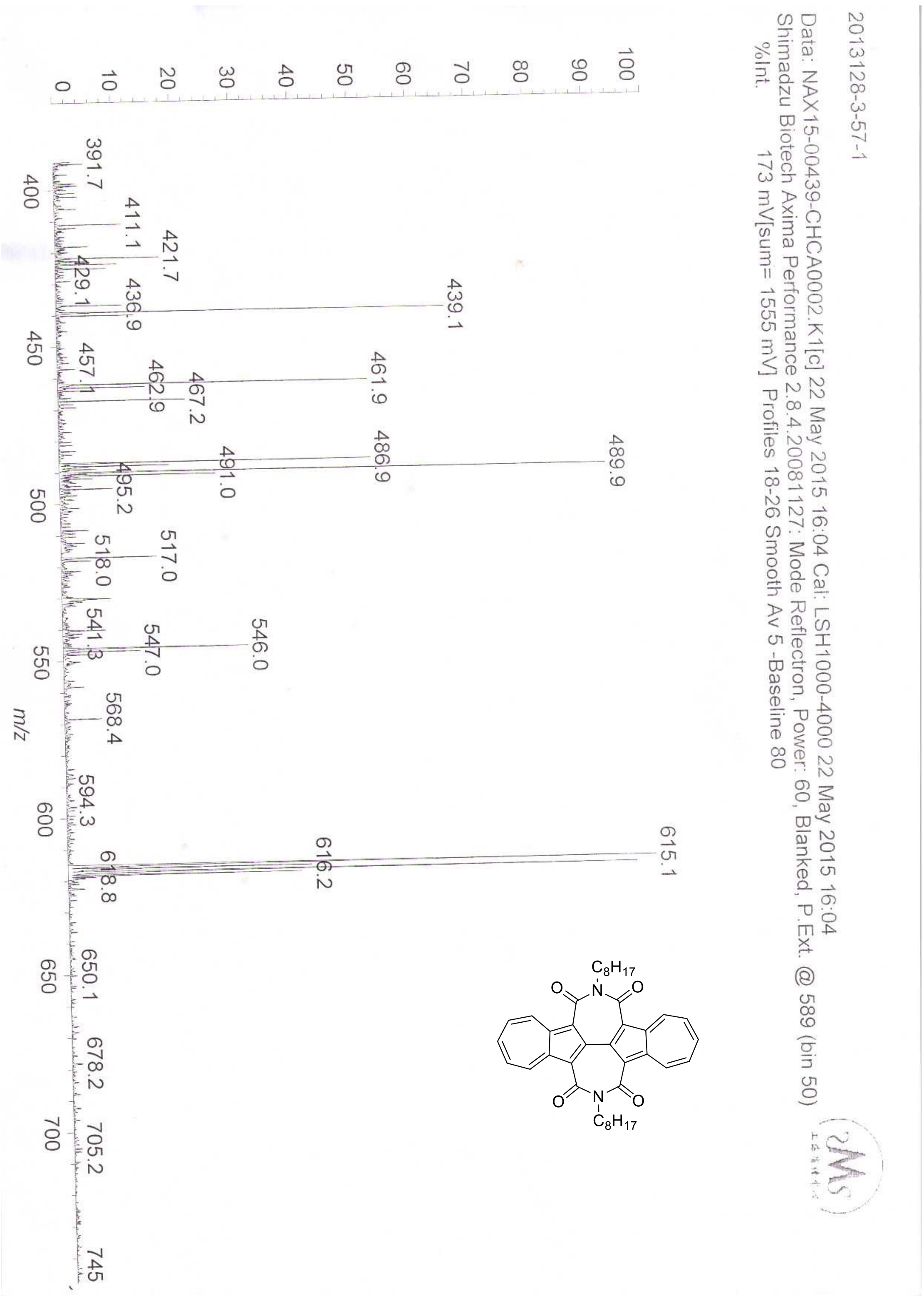

Figure S30. MS spectrum of **BAzDI-1**

National Center for Organic Mass Spectrometry in Shanghai
Shanghai Institute of Organic Chemistry
Chinese Academic of Sciences
High Resolution MS DATA REPORT

Instrument: Thermo Fisher Scientific LTQ FT Ultra

Card Serial Number : M152095

Sample Serial Number: 3-57-1

Operator : HUAQIN Date: 2015/06/18

Operation Mode: DART Postive

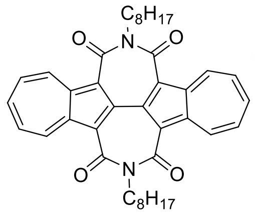

Elemental composition search on mass 617.34

m/z= 612.34-622.34

m/z	Theo. Mass	Delta (ppm)	RDB equiv.	Composition
617.3362	617.3360	0.26	20.0	$C_{38}H_{43}O_3N_5$
	617.3365	-0.49	10.5	$C_{31}H_{49}O_7N_4Si$
	617.3366	-0.57	2.0	$C_{25}H_{51}O_{14}N_3$
	617.3352	1.68	5.5	$C_{30}H_{53}O_{11}Si$
	617.3374	-1.92	19.5	$C_{40}H_{45}O_4N_2$
	617.3347	2.42	15.0	$C_{37}H_{47}O_7N$
	617.3378	-2.67	10.0	$C_{33}H_{51}O_8NSi$
	617.3379	-2.75	1.5	$C_{27}H_{53}O_{15}$
	617.3338	3.85	6.0	$C_{28}H_{51}O_{10}N_3Si$
	617.3334	4.60	15.5	$C_{35}H_{45}O_6N_4$

Figure S31. HRMS spectrum of **BAzDI-1**

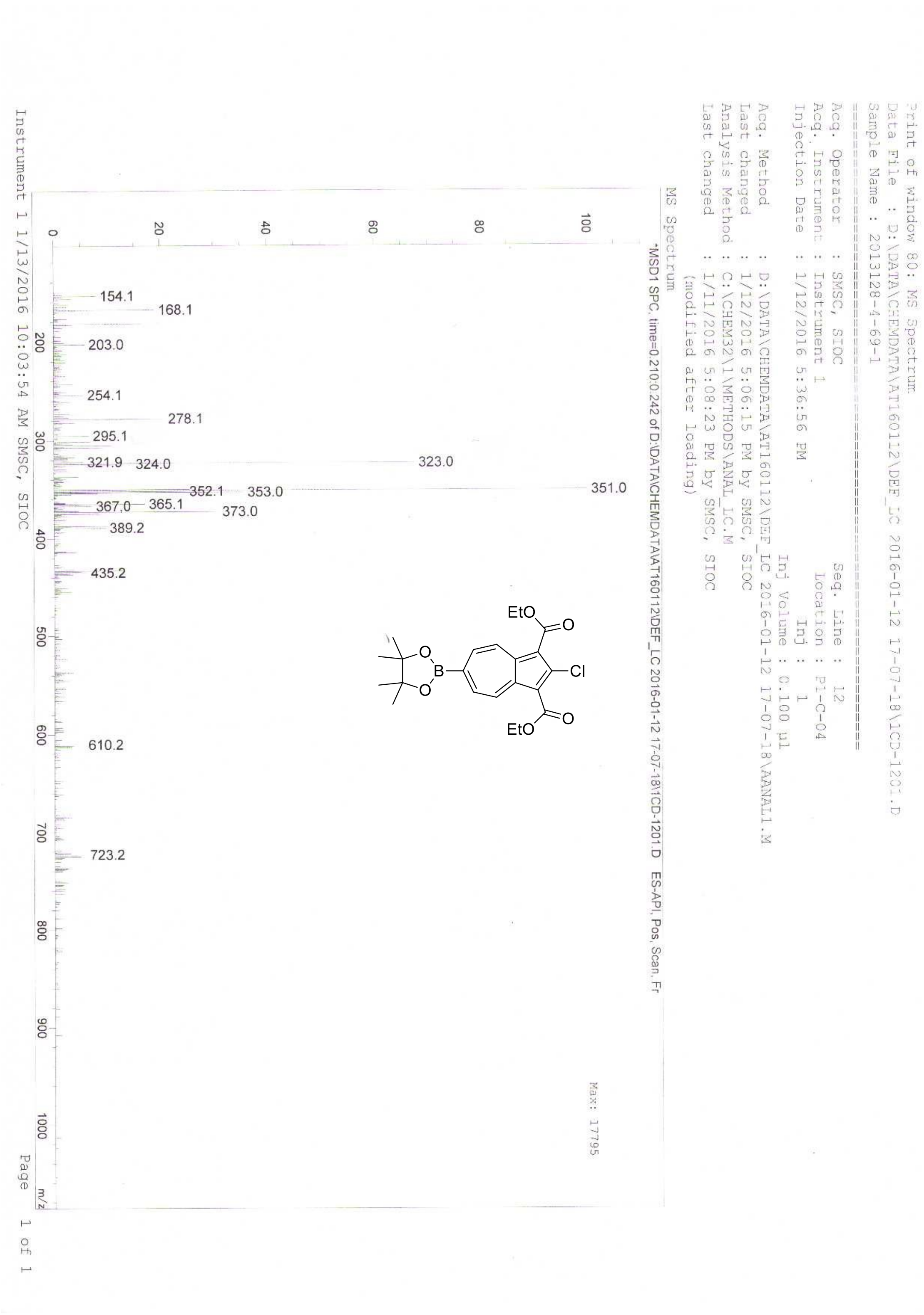

Figure S32. MS spectrum of **7**

Shanghai Institute of Organic Chemistry
Chinese Academic of Sciences
High Resolution MS DATA REPORT

Instrument: Thermo Fisher Scientific LTQ FT Ultra

Card Serial Number : D160858

Sample Serial Number: 4-69-1

Operator :HUAQIN Date: 2016/03/31

Operation Mode: DART Positive

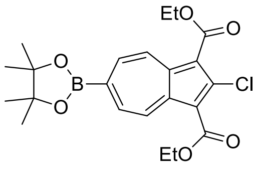

Elemental composition search on mass 432.16

m/z= 427.16-437.16

m/z	Theo. Mass	Delta (ppm)	RDB equiv.	Composition
432.1616	432.1620	-1.03	9.0	$C_{22}H_{27}O_6{}^{10}BCl$
	432.1606	2.23	5.5	$C_{20}H_{31}O_5NClS$
	432.1628	-2.85	14.5	$C_{26}H_{26}O_3NS$
	432.1601	3.35	10.0	$C_{23}H_{28}O_6S$
	432.1595	4.76	13.0	$C_{26}H_{27}O{}^{10}BClS$
	432.1594	4.95	19.5	$C_{29}H_{22}O_3N$

Figure S33. HRMS spectrum of **7**

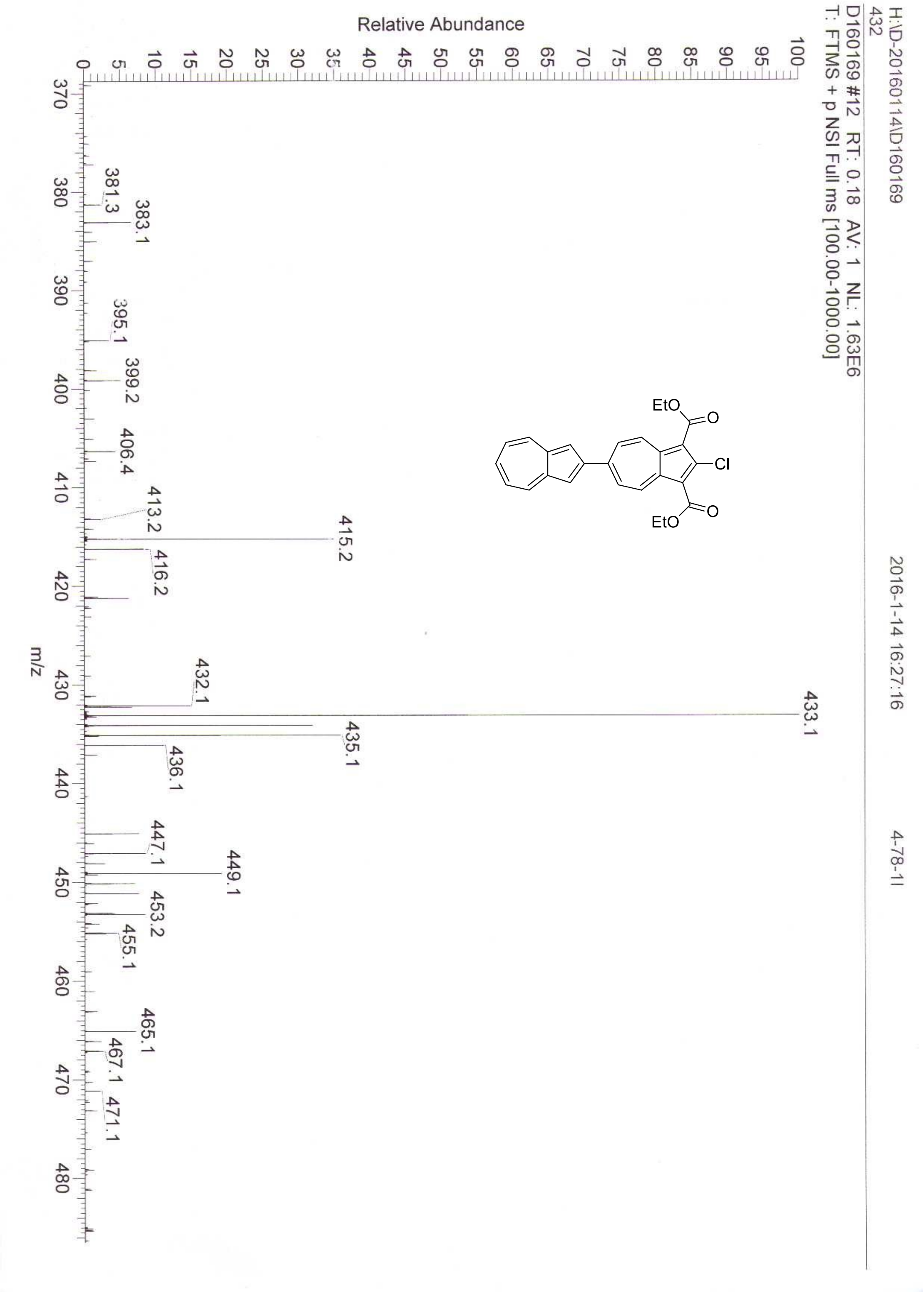

Figure S34. MS spectrum of **9**

Shanghai Institute of Organic Chemistry
Chinese Academic of Sciences
High Resolution MS DATA REPORT

Instrument: Thermo Fisher Scientific LTQ FT Ultra

Card Serial Number : D160170

Sample Serial Number: 4-78-1

Operator : HUAQIN Date: 2016/01/14

Operation Mode: DART-Positive

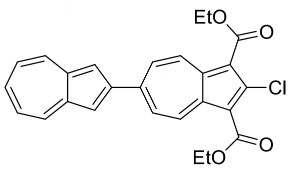

Elemental composition search on mass 433.12

m/z= 428.12-438.12

m/z	Theo. Mass	Delta (ppm)	RDB equiv.	Composition
433.1201	433.1201	-0.08	15.5	$C_{26}H_{22}O_4Cl$
	433.1210	-2.04	25.0	$C_{30}H_{15}ON_3$
	433.1188	3.02	16.0	$C_{24}H_{20}O_3N_3Cl$
	433.1215	-3.17	20.5	$C_{27}H_{18}N_4Cl$
	433.1183	4.15	20.5	$C_{27}H_{17}O_4N_2$

Figure S35. HRMS spectrum of **9**

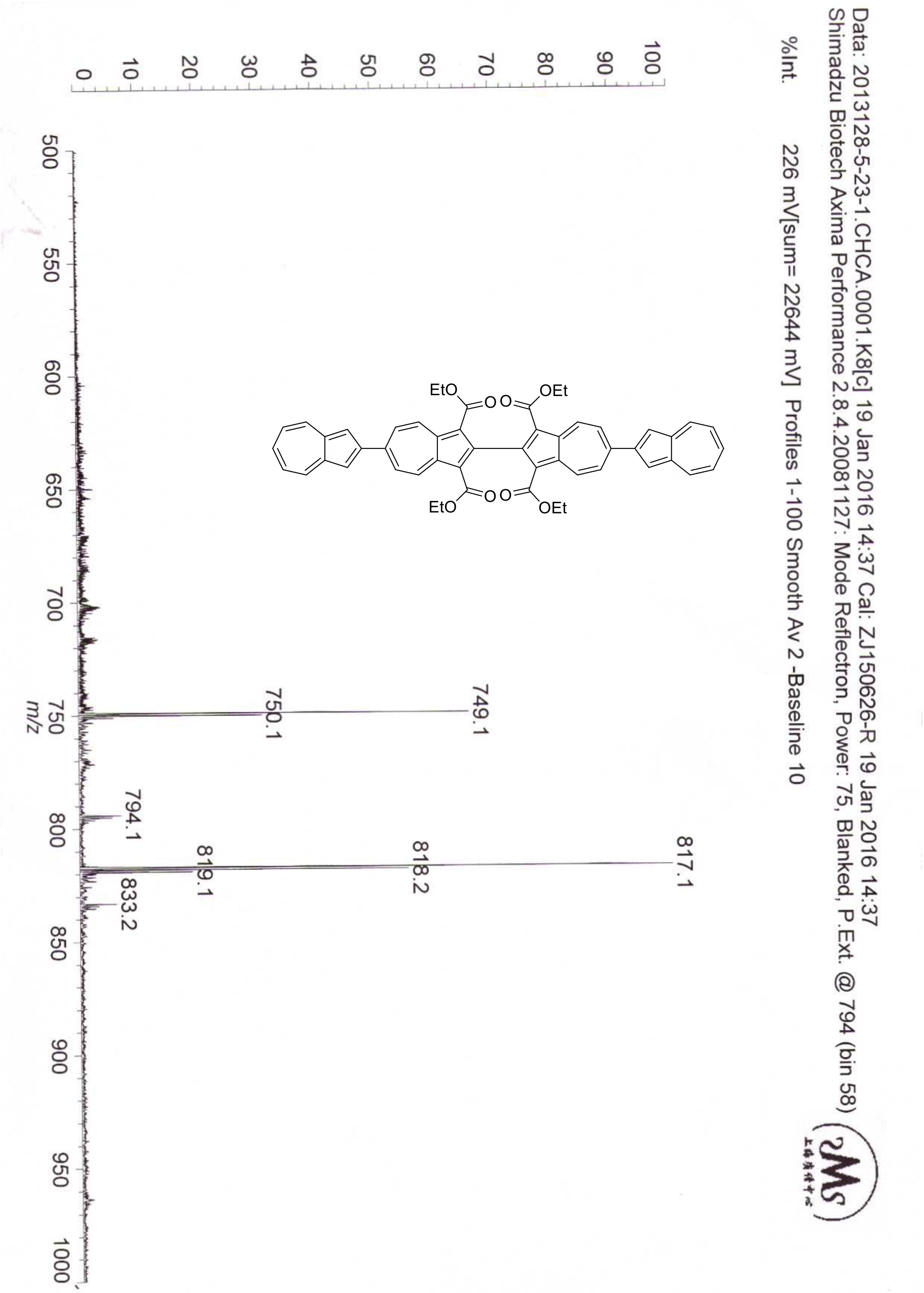

Figure S36. MS spectrum of **10**

Shanghai Institute of Organic Chemistry
Chinese Academic of Sciences
High Resolution MS DATA REPORT

Instrument: Thermo Fisher Scientific LTQ FT Ultra

Card Serial Number : D160862

Sample Serial Number: 5-23-1

Operator :HUAQIN Date: 2016/03/31

Operation Mode: DART Positive

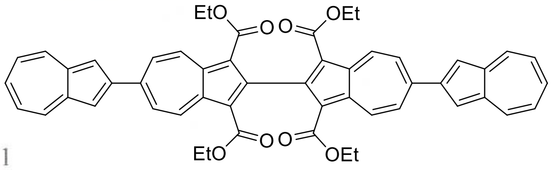

Elemental composition search on mass 795.29

m/z= 790.29-800.29

m/z	Theo. Mass	Delta (ppm)	RDB equiv.	Composition
795.2945	795.2952	-0.99	31.5	$C_{52}H_{43}O_8$
	795.2961	-2.08	30.5	$C_{53}H_{47}O_3S_2$
	795.2979	-4.36	36.0	$C_{55}H_{41}O_5N$

Figure S37. HRMS spectrum of **10**

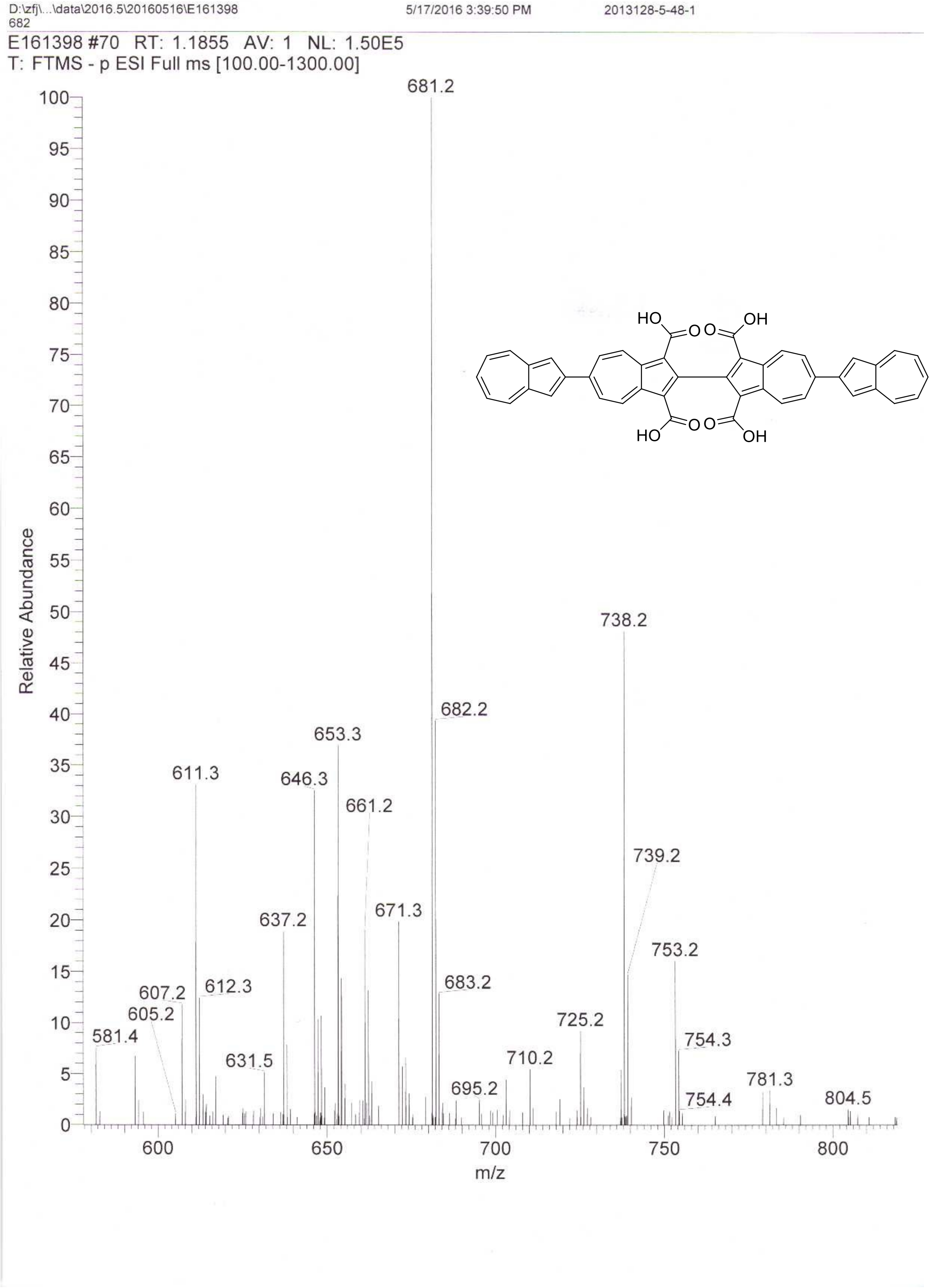

Figure S38. MS spectrum of **11**

National Center for Organic Mass Spectrometry in Shanghai
Shanghai Institute of Organic Chemistry
Chinese Academic of Sciences
High Resolution MS DATA REPORT

Instrument: Thermo Fisher Scientific LTQ FT Ultra

Card Serial Number : E161398

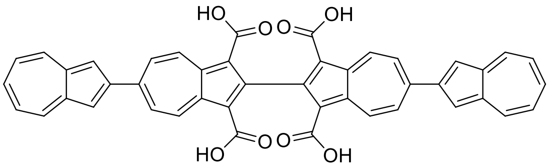

Sample Serial Number: 2013128-5-48-1

Operator : ZHUFJ Date: 2016/05/16

Operation Mode: ESI Negative

Elemental composition search on mass 681.15

m/z= 676.15-686.15

m/z	Theo. Mass	Delta (ppm)	RDB equiv.	Composition
681.1544	681.1555	-1.57	32.5	$C_{44}H_{25}O_8$
	681.1573	-4.29	19.5	$C_{32}H_{29}O_{15}N_2$
	681.1515	4.33	28.5	$C_{39}H_{25}O_{10}N_2$

Figure S39. HRMS spectrum of **11**

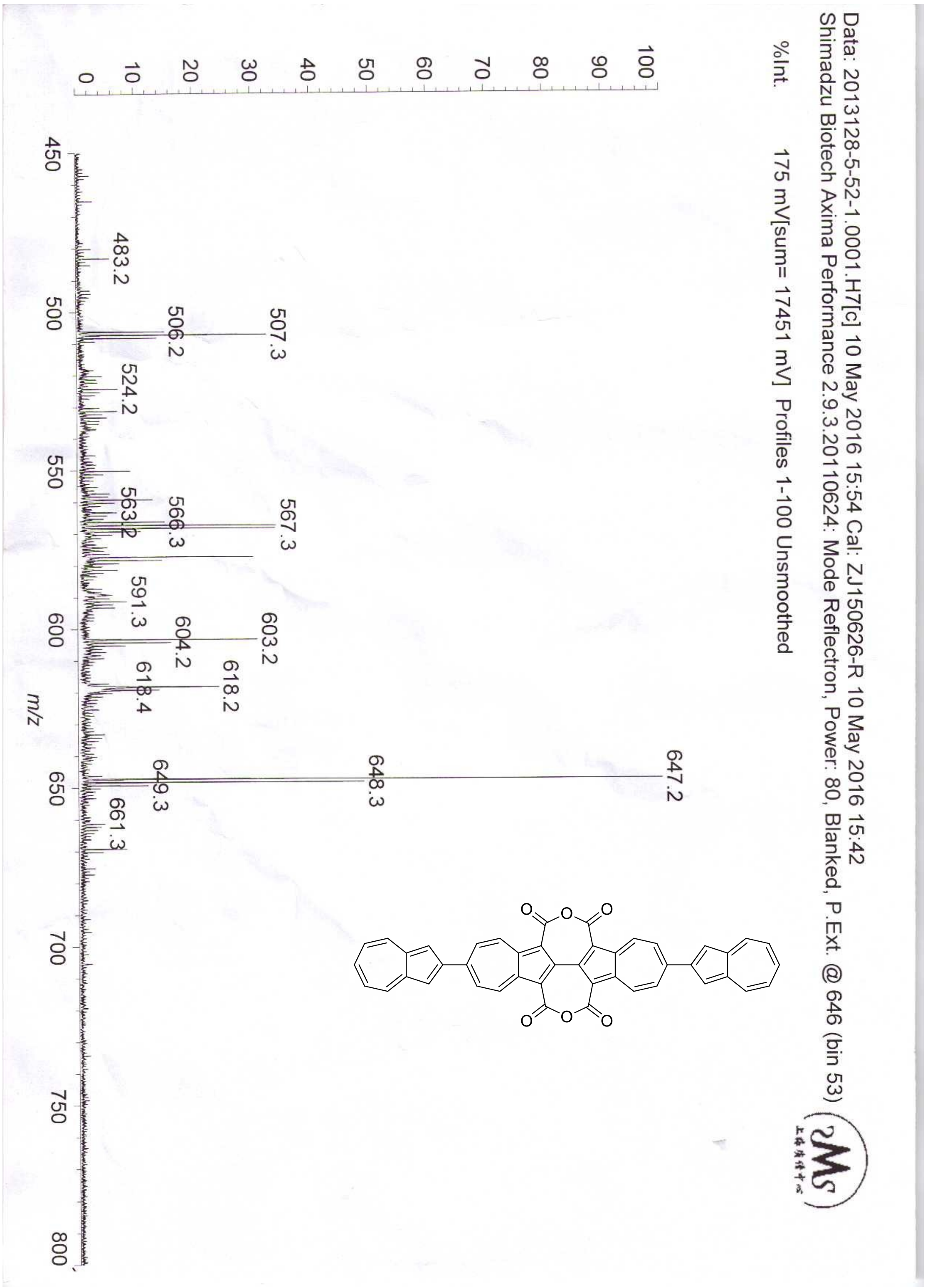

Figure S40. MS spectrum of **12**

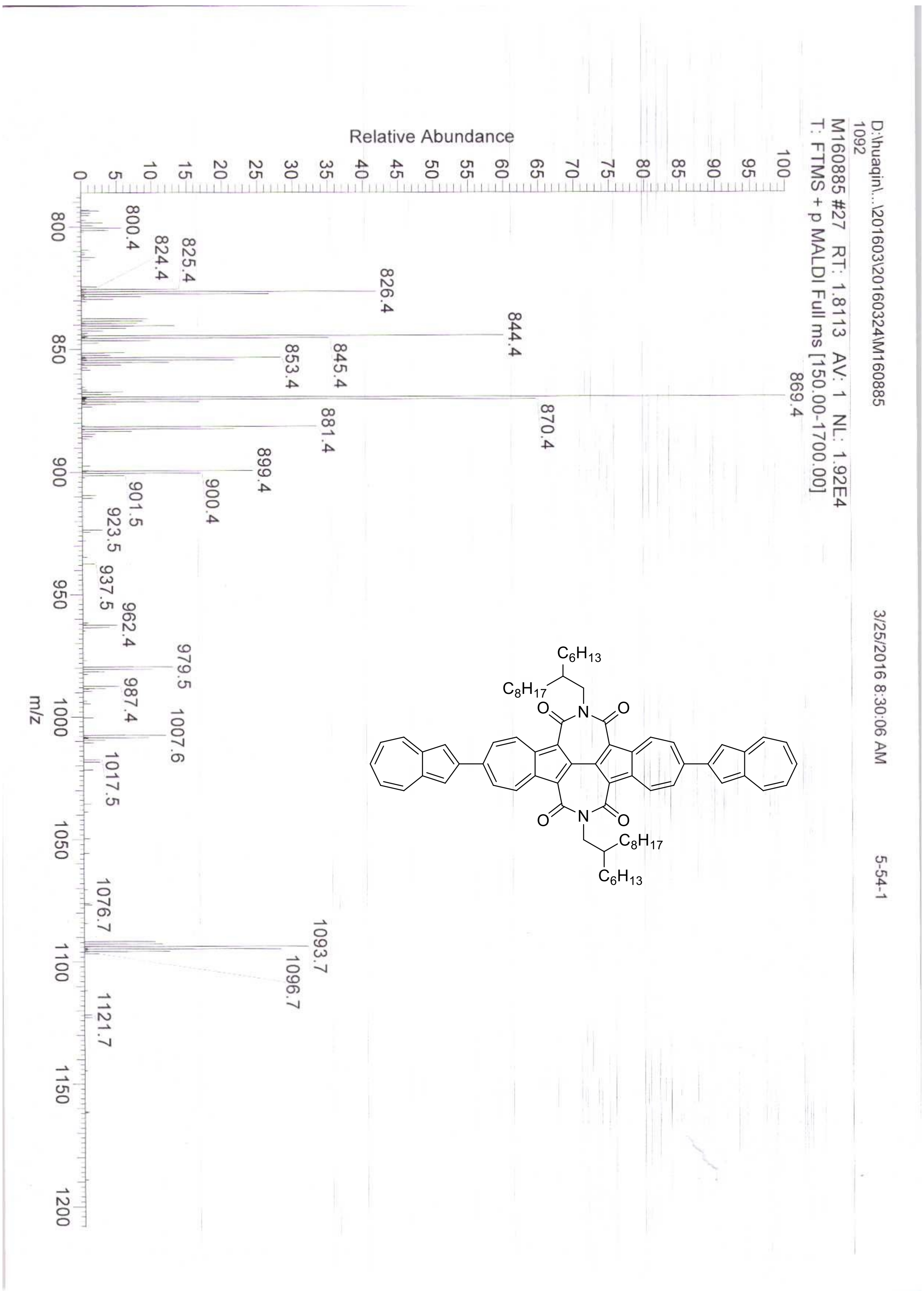

Figure S41. MS spectrum of **BAzDI-2**

National Center for Organic Mass Spectrometry in Shanghai
Shanghai Institute of Organic Chemistry
Chinese Academic of Sciences
High Resolution MS DATA REPORT

Instrument: Thermo Fisher Scientific LTQ FT Ultra

Card Serial Number : M160886

Sample Serial Number: 5-54-1

Operator : HUAQIN Date: 2016/03/24

Operation Mode: MALDI-FT_DHB

Elemental composition search on mass 1093.68

m/z= 1088.68-1098.68

m/z	Theo. Mass	Delta (ppm)	RDB equiv.	Composition
1093.6812	1093.6817	-0.43	33.5	$C_{76}H_{89}O_4N_2$
	1093.6803	0.79	34.0	$C_{74}H_{87}O_3N_5$
	1093.6844	-2.89	38.0	$C_{79}H_{87}ON_3$

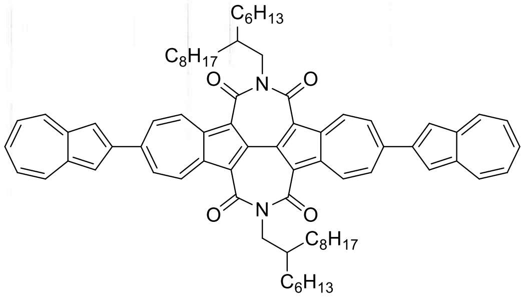

Figure S42. HRMS spectrum of **BAzDI-2**

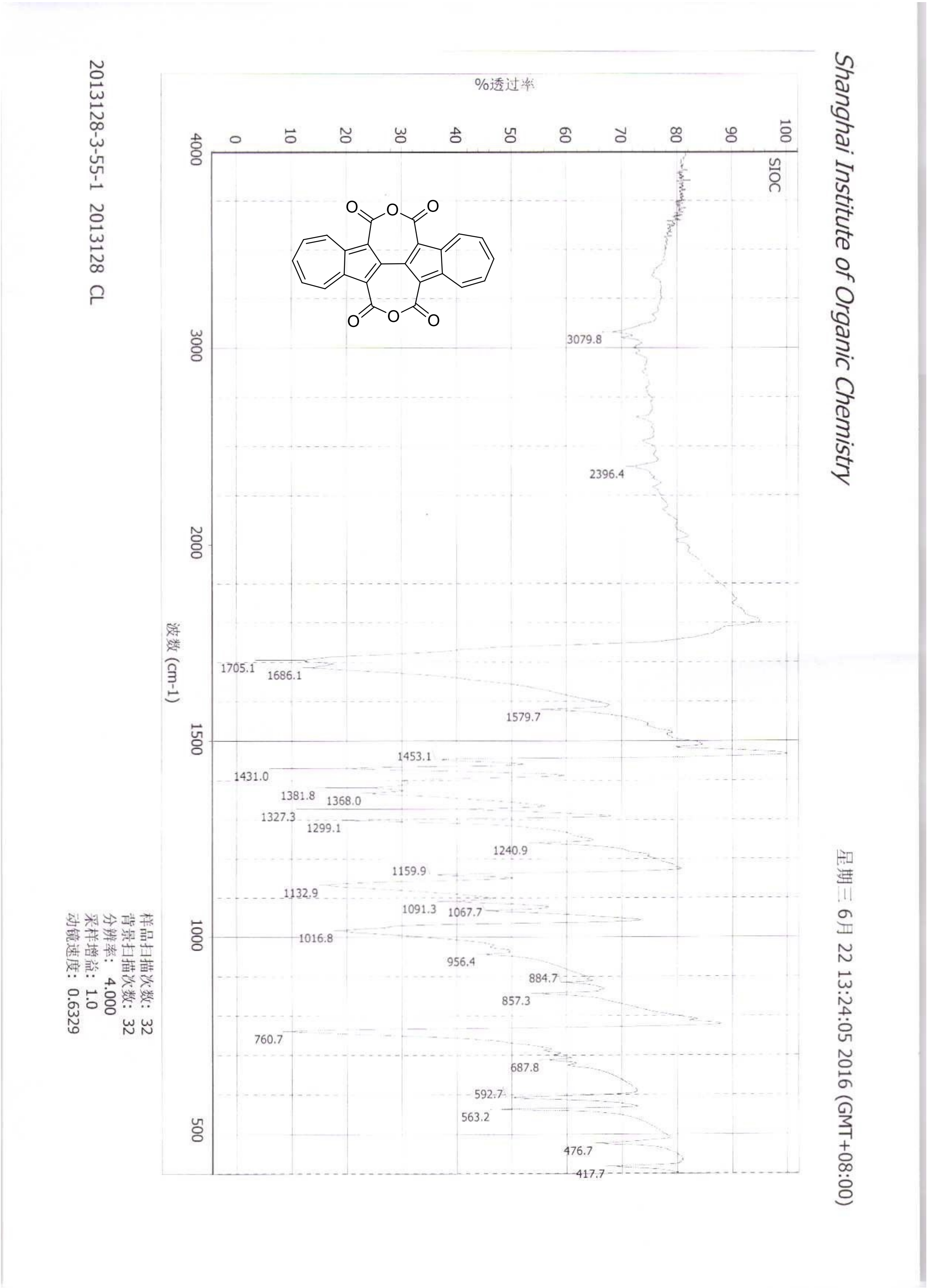

Figure S43. IR spectrum of **4**

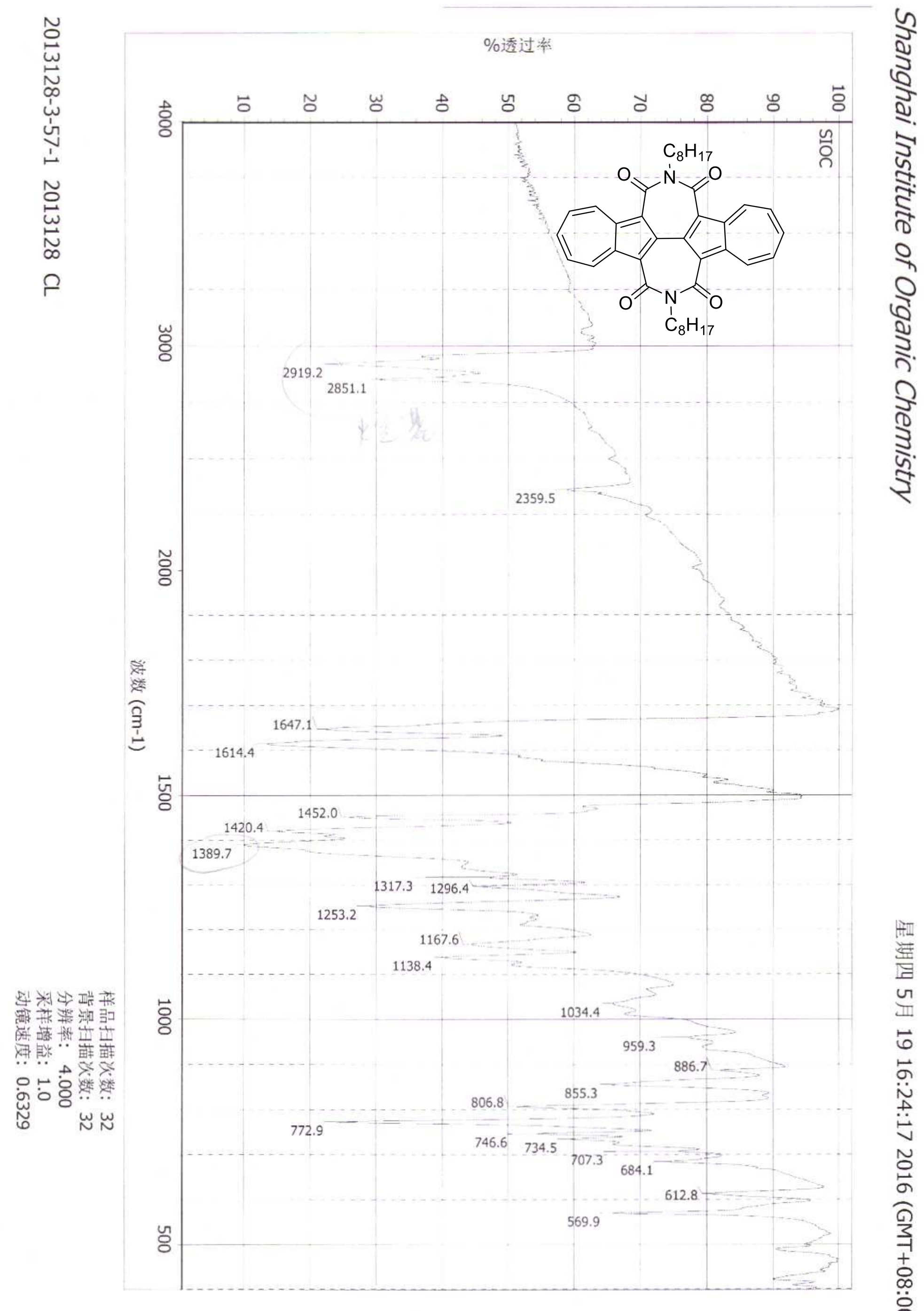

Figure S44. IR spectrum of
BAzDI-1

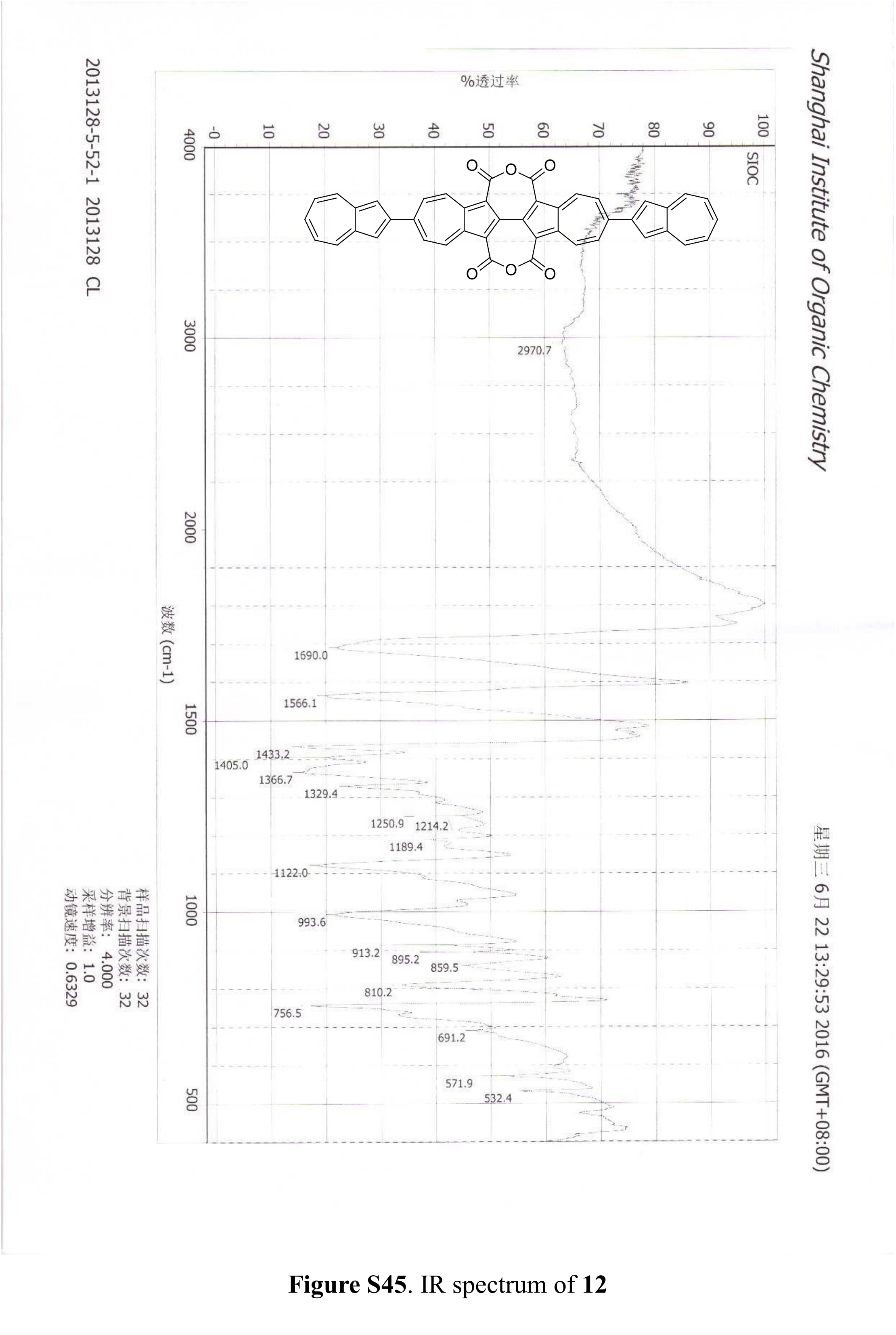

Figure S45. IR spectrum of **12**

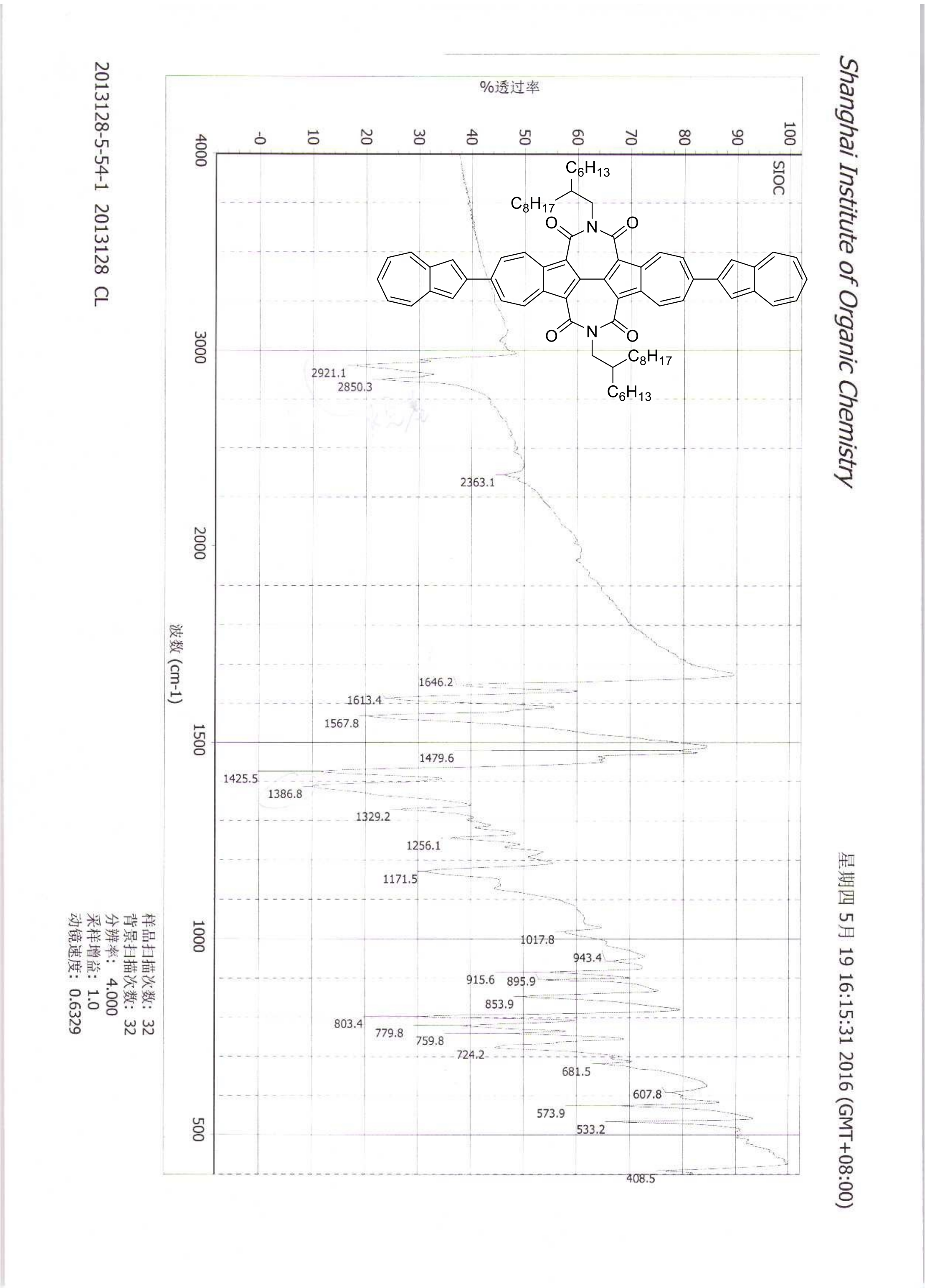

Figure S46. IR spectrum of **BAzDI-2**
